# New Insights Into Mouthings: Evidence From a Corpus-Based Study of Russian Sign Language

**DOI:** 10.3389/fpsyg.2021.779958

**Published:** 2022-02-22

**Authors:** Anastasia Bauer, Masha Kyuseva

**Affiliations:** ^1^Slavic Department, University of Cologne, Cologne, Germany; ^2^School of Literature and Languages, University of Surrey, Guildford, United Kingdom

**Keywords:** mouthings, corpus analysis, Russian Sign Language, multimodality, language contact phenomenon, mouth actions

## Abstract

While some aspects of mouthings have been previously investigated, many topics in the use of this cross-modal contact phenomenon in sign languages remain un(der)studied, and not much is known about mouthings in Russian Sign Language (RSL), in particular. This article examines various aspects of mouthings as these are used by native RSL signers and aims to contribute new insights into the use and origin of mouthings in this sign language. Based on novel data from the online RSL Corpus alongside additional elicited data, we describe the distribution, forms, functions and spreading patterns of mouthings. Our findings furthermore show that sign languages exhibit more extensive variation in the use of mouthings than has previously been thought. Moreover, we – thus far uniquely – describe mouthings also as a written-language-based contact phenomenon. This study has the potential to provide a better understanding of the nature of such contact-induced features as mouthings in sign languages in general and reveals a complex interplay of the modalities of signed, spoken and written languages.

## Introduction

The focus of the present study is on mouthings, that is, on mouth actions that resemble the articulation of spoken language words (Boyes-Braem and Sutton-Spence, 2001). For instance, while producing the sign WATER in Russian Sign Language (henceforth, RSL), the signer articulates (a part of) the Russian word *voda* ‘water’. These mouth actions have to be differentiated from mouth actions that have nothing to do with words from the surrounding spoken language. As an example of the latter, when producing the RSL sign CAN, the signer usually closes their mouth as if making the sound [ap] (Kyuseva, 2020, p. 8). This type of mouth action is called a mouth gesture (Boyes-Braem, 2001). Mouth gestures are different from mouthings and are considered to be a sign language inherent category. In the majority of their uses, they either constitute a nearly obligatory semantically empty component of a sign (as in the example CAN above), or convey an adjectival (e.g., ‘thick’ or ‘thin’) or adverbial (e.g., ‘intensely’) semantic information additional to that specified by the manual sign(s) (Crasborn et al., 2008).

Mouthings have been a subject of linguistic research for over 30 years now (see the pioneering works of Vogt-Svendsen, 1981; Schermer, 1990). Present in nearly all studied sign languages (with one reported exception in the case of Kata Kolok; see de Vos and Zeshan, 2012), mouthings contribute significantly to the formal and semantic aspects of these languages. By studying them, we can gain a special window onto what goes on in the mind of the signer when producing and processing sign language (Vinson et al., 2010).

Even though studies attest that mouthings comprise the largest category of all mouth actions (Crasborn et al., 2008), their linguistic role, their functions in discourse, their spreading patterns and the principles governing their occurrence in native signing have still not been thoroughly investigated. We still lack descriptions of the mouthings in a great number of sign languages, including in RSL. Only a few Deaf^1^ community sign languages, such as especially the British (BSL), Australian (Auslan), Swedish (STS), German (DGS), and Dutch (NGT) ones, alongside a small number of other, mostly European sign languages, have been analyzed with respect to their mouthings (Ebbinghaus and Heßmann, 1995; Sutton-Spence and Day, 2001; Bank et al., 2011; Mohr, 2014; Johnston et al., 2016; Mesch and Schönström, 2021). Although, so far, most sign language research has focused on ASL, it is interesting to note that the vast bulk of the research carried out on mouthing has been for European sign languages, while ASL researchers have largely ignored mouthing for many years (an exception is Nadolske and Rosenstock, 2008).

This paper contributes to the field by presenting the first detailed corpus-based description of mouthings in RSL. The paper is structured as follows. Section “Research on Mouthings in Sign Languages” establishes the necessary background on research into mouthings in different sign languages; Section “Russian Sign Language” provides sociolinguistic information about RSL. Section “Materials and Methods” discusses the methodology adopted in this study and introduces the three research questions that we pose. These questions are subsequently answered in sections “New insights into the distribution patterns of mouthings,” “New insights into the functions of mouthings,” and “New insights into the origin of mouthings.” Specifically, section “New insights into the distribution patterns of mouthings” investigates the form and distribution of mouthings, section “New insights into the functions of mouthings” explores the functions of mouthings, and section “New insights into the origin of mouthings” deals with the origin of mouthings. The paper concludes with section “Discussion,” which presents a discussion of the implications that the analyzed data have for linguistic typology and theoretical accounts of mouthings.

### Research on Mouthings in Sign Languages

Several authors have explored the phenomenon of mouthing using data from various sign languages, yet mouthing patterns and their grammatical functions are still not fully understood. After the two pioneering studies on mouthings, namely in Norwegian Sign Language (Vogt-Svendsen, 1981) and in NGT (Schermer, 1990), a book edited by Boyes-Braem and Sutton-Spence (2001) laid the groundwork for further research into this unique phenomenon. The book standardized the terminology ‘mouthings’ and ‘mouth gestures’ for two different types of mouth actions and brought together papers on mouthings in nine different sign languages (Ajello et al., 2001; Bergmann and Boyes-Braem, 2001; Bergman and Wallin, 2001; Ebbinghaus and Heßmann, 2001; Rainò, 2001; Schermer, 2001; Sutton-Spence and Day, 2001; Vogt-Svendsen, 2001; Zeshan, 2001). Recently, there have also been four thorough corpus studies on mouthings in Irish Sign Language, NGT, Auslan and Hungarian Sign Language (Bank, 2014; Mohr, 2014; Johnston et al., 2016; Racz-Engelhardt, 2016).

Boyes-Braem and Sutton-Spence (2001) have defined mouthings as silent articulations of words from the surrounding spoken language. Correspondingly, it is widely accepted among linguists that mouthings originated as borrowings through imitation of the lip movements that are made when pronouncing words of the surrounding spoken language.

Mouthings are omnipresent in deaf native signing (Bank, 2014). A DGS corpus study revealed that more than 80% of all utterances involved at least one mouthing. That is to say that every second manual element in a typical signed utterance in DGS is accompanied by a mouthing (Ebbinghaus and Heßmann, 1995). In the Auslan data, more than 70% of all mouth actions were mouthings and, in the NGT data, 80% of mouth actions were mouthings. The NGT and Auslan corpora are similar in the genres they encompass and are well suited for comparison with the RSL corpus (Bank, 2014; Johnston et al., 2016).

There is quite some debate in the sign language literature about the linguistic status of mouthings. A disputed question is whether mouthings are constitutive elements of sign languages or instances of code mixing (see Bauer, 2019 for an overview). The opinions range in terms of ascribed status from mouthings as part of a sign’s phonological description in line with the other phonological formational categories of hand configuration, location and movement (Ajello et al., 2001; Bergman and Wallin, 2001; Boyes-Braem, 2001; Rainò, 2001; Sutton-Spence and Day, 2001; Vogt-Svendsen, 2001; Crasborn et al., 2008; Hosemann, 2015; Steinbach, 2016) to mouthings as instances of online code-blending, where signers can freely and simultaneously combine elements from a spoken and signed language (Ebbinghaus and Heßmann, 2001; Hohenberger and Happ, 2001; Vinson et al., 2010; Bank et al., 2011, 2016; Johnston et al., 2016; Giustolisi et al., 2017; Perniss et al., 2020). Many studies have contributed to this debate and there are good arguments in favor of each view. The question of the linguistic status of mouthings thus remains unresolved, and we doubt that it will be definitively answered in the near future.

In this study, we treat mouthings as integrally constitutive of sign language use and a result of complex processes of cross-modal language contact (cf. Capek et al., 2008). We observe that mouthings are perceived by deaf native signers in monolingual contexts as a necessary and vital part of their language (also in ISL by Mohr, 2012, 2014). The metalinguistic awareness study by Fontana and Ferrara (2019) has demonstrated that signing without mouthings is interpreted by native signers as being non-fluent and inauthentic. We believe there are important questions that need to be resolved first, namely: how are mouthings used in signed discourse (what are the manual forms they occur with and which form do they have in which contexts: reduced, inflected, spread etc.) and how do they contribute to the overall meaning of a signed construction (i.e. what functions do mouthings perform)? Therefore, the analysis presented in this paper deliberately stays outside of the debate on the linguistic status of mouthing and does not aim to contribute to this scholarly discussion.

#### Mouthing Forms

In terms of form, mouthings may be regarded as standard, fully articulated, inflected, temporally reduced or spread across neighboring manual signs. Mouthings that are time-aligned with, and have a similar meaning to, the signs they accompany are known as standard mouthings (Bank et al., 2011; Bank, 2014).

Mouthings are typically observed in their uninflected citation forms (Boyes-Braem, 2001; Hohenberger and Happ, 2001). However, some studies have reported on the occurrence of inflected mouthings with examples of tense marking on verbs or plural marking on nouns (Mohr, 2014; Racz-Engelhardt, 2016). Bauer (2019), moreover, has shown that mouthings in RSL can be inflected for case, gender, number, and aspect.

A mouthing is considered to be reduced when only some of its parts are visible, as in the DGS examples *wi(chtig*) ‘important,’ *fer(tig)* ‘ready,’ NGT *aksp* ‘accepteren = to accept’ or RSL *sob(aka*) ‘dog’ or *Novosib(irsk)* (Boyes-Braem, 2001, p. 104; Bank, 2014, p. 24; Bauer, 2019, p. 22).^2^ Not much research has been done on the use of reduced mouthings in different sign languages. Mesch and Schönström (2021) have shown that reduced mouthings are used much more often by deaf L1 signers than by L2 learners of Swedish Sign Language (STS). L2 signers tend to use full mouthings while signing. Ebbinghaus and Heßmann (1995) observed that, in DGS, reductions occurred more often for verbs than for nouns. They assume that a reduction of a German verb does not impair the understanding of the meaning in the same way that the reduction of a noun can. Johnston et al. (2016, p. 21) reported that 95% of mouthings in the Auslan corpus were fully articulated and less than 4% were reduced. The results for NGT appear to be mixed. While some signs, such as TALK, appear to be reduced in 80% of instances in the NGT corpus, other signs (e.g. PREVIOUS or HEARING) show a preference for the full two-syllable citation form (Bank et al., 2011, p. 261). What all reduced mouthings appear to have in common is that they are overwhelmingly initial segments of a lexical word of the surrounding spoken language. This means that only the first part of the corresponding word is visible^3^, as shown in (1–2). Mouthings may even be reduced to a single syllable or to a very short mouth movement mimicking articulation of only the first sound, as observed in RSL mouthings for *ždu* ‘wait’ or *babuška* ‘grandmother’ reduced to only ž or *b* respectively (also found in ISL, as reported by Mohr and Leeson, in press).

Sandler and Lillo-Martin (2006, p. 105) have argued that reduced mouthings conform to the rhythm of the monosyllabic form of the co-occurring manual sign, while Bank et al. (2011) have shown that unstressed syllables are reduced more often than are stressed ones. All mouthings in the latter study’s analyzed NGT data contained stressed syllables. The authors therefore suggest that temporal reduction typically happens in the form of deleting word-final consonants, while reasoning that signers have knowledge of the rhythmic structure of Dutch words (ibid.: 264). Although the authors did not give a detailed explanation of how this happens, the hypothesis appears to be plausible. Stressed syllables are usually longer and more strongly articulated and with less vowel reduction than are unstressed syllables (Grice and Baumann, 2007). This entails that the stressed part of the spoken-language item is the most visible one to the interlocutor during lip reading. By recognizing the stressed syllable, signers can perceive the rhythmic structure of spoken words. Given this state of affairs, the phenomenon of reduced mouthings calls for more research. RSL lends itself well to verification of the stressed syllable hypothesis by Bank et al. (2011). In Russian, the stress can occur in various positions within a word. This means that the stress is distinctive, i.e. two words can be distinguished based only on their stress pattern. Additionally, due to the vowel reduction phenomenon in spoken Russian, vowel quality varies greatly depending on whether a vowel occurs in a stressed or an unstressed syllable (Yanushevskaya and Bunčić, 2015). Section “New insights into the origin of mouthings” of this article evaluates the above claims and gives answers as to which parts of the respective spoken words are typically articulated in RSL mouthings and explains how this finding may yield insight into the origin of mouthings.

A mouthing is considered to be spread when its duration extends over more than one manual sign. Research on spreading revolves around the following topics: the direction of spreading, the scope of spreading, the source and goal signs of spreading and the functions of spreading. In terms of direction, spreading can be progressive or regressive. Progressive spreading starts together with the semantically congruent sign and extends over the next manual sign (or signs). Regressive spreading starts before the semantically congruent sign and then extends over it. See example (1), with progressive, and (2), with regressive spreading, below:


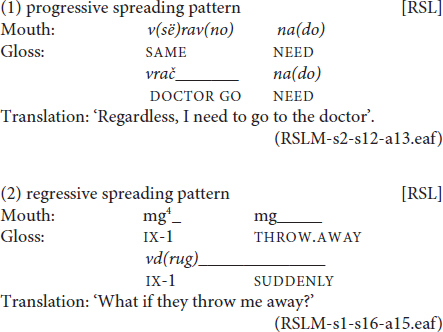


In (1), the mouthing of *vrač* ‘doctor’ extends over both its designated manual counterpart and the next classifier predicate ‘go.’ In (2), the mouthing of *vdrug* ‘suddenly’ begins during a pointing sign and extends over the following sign, its manual counterpart SUDDENLY. Languages have been reported to differ with respect to the direction of spreading they prefer. Crasborn et al. (2008) showed that British Sign Language (BSL) and NGT abide by the progressive pattern, while STS allows for both directions.

In terms of scope, a mouthing can extend over one or more additional signs. Existing research suggests that, although spreading over several signs is possible, spreading over just one additional sign is much more frequent. For example, out of 810 spreadings in the NGT corpus, only six percent are spread over several signs, while 94 percent are spread over a single immediately adjacent sign (Bank, 2014).

Irrespective of the scope and direction of spreading, mouthing usually extends from nouns, verbs, adverbs and adjectives to pointing signs/indexes, possessive pronouns, determiners, classifiers, palm-up and prepositions (Crasborn et al., 2008). In other words, the starting point signs tend to be content words, and the end point signs tend to be functional words. Pointing signs are the most frequent end point signs in spreading, which is probably due to the fact that they are prosodically light and consist of a simple articulatory representation (Sandler, 1999). Crasborn et al. (2008) discussed an exception to this generalization and provided an example in SSL where a mouthing is spread from one content word over another content word. In their example, the mouthing of *mål*, paired to the nominal sign M̊AL ‘goal,’ is spread rightward over the noun LINJE ‘line,’ thus constituting a morphological compound MÅALLINJE ‘finish line’. The RSL data presented here will expand the list of exceptions and show interesting examples, such as where a mouthing is spread from a function word to a content word (section “Spreading Patterns”). We will argue that this pattern has a specific function associated with it, which will be discussed in the next section.

#### Mouthing Functions

Mouthings appear to play an important role in facilitating understanding in sign languages and are known to have various grammatical, lexical, prosodic, stylistic and sociolinguistic functions (Boyes-Braem, 2001; Mohr, 2014; Safar et al., 2015).

In most cases, mouthings correspond exactly to a manual sign both in terms of temporal alignment and semantic congruency. This semantically congruent type of mouthing, the standard mouthing, is the most frequent one (Schermer, 1990; Boyes-Braem, 2001; Bank et al., 2011). It is illustrated by the RSL example in (3).


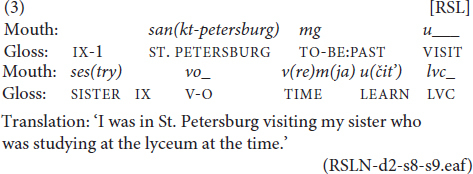


In (3), the mouthed parts of the words *san(kt-petesrburg)*, *ses(tra)* ‘sister’ and *u(čit’)* ‘to learn’ have the same meaning as the manual signs they co-occur with. Mouthing can also add meaning to a sign by indicating a more specific reading of it, as, for example, in the DGS sign SIBLING with the German mouthings *Schwester* ‘sister’ or *Bruder* ‘brother’. Such simultaneous mouthings seem to be obligatory in order to disambiguate or further specify the sign in question (Boyes-Braem, 2001). Examples of RSL polysemous signs that are disambiguated by mouthing are NEUDOBNO ‘uncomfortable’ versus NEVKUSNO ‘not tasty,’ and KOŠKA ‘cat’ versus LASKA ‘tenderness’ (see also the examples in section “Mouthing and Degrees of Lexicalization”). Such cases are rare in the RSL corpus.

Mouthing appears to fulfill a word-class marking function in sign languages. Mouthings are reported to accompany nouns and morphologically simpler signs more frequently than they do verbs or morphologically more complex signs (such as classifiers) (Kimmelman, 2009). In the studies of noun–verb pairs in Austrian Sign Language (ÖGS) and Auslan, it was noticed that mouthing is much more likely to occur with nouns than with verbs. In Hunger’s (2006) study of ÖGS, 92% percent of nouns and only 52% of verbs were accompanied by mouthing. In Auslan, about 70% of the nouns studied were accompanied by mouthing, whereas only 13% of the verbs were (Johnston and Schembri, 2007). Bank et al. (2011), however, found no word-class specific pattern in their study of NGT mouthings.

The spreading of a mouthing is reported to serve as one of the building blocks of the prosodic structure of sign language phrases. Sandler (1999) has argued that mouthing can bind a host content word and a cliticized pointing sign to form a prosodic phrase. Since the prosodic structure is believed to reveal part of the invisible syntactic structure, some examples of mouthing spreading can be analyzed as instances of syntactic binding. For instance, Boyes-Braem (2001) provided examples wherein mouthings bind constituents of a noun phrase, as well as verbs with subjects in Swiss German Sign Language. Crasborn et al. (2008) gave examples of verb–adverb combinations (e.g. ‘lay silent’), verb–object clusters (e.g. ‘see field’) and nominal compounds in SSL. The RSL data presented in this paper will reveal another function of mouthing that has not been discussed before. We call it the “discourse function,” since it is connected with the phenomenon of turn-taking.

#### Summary

As we have seen, mouthings represent a ubiquitous yet heterogenous phenomenon within the world’s sign languages. They occur in standard, fully articulated and inflected forms and may be temporally reduced or spread across neighboring manual signs. Their functions range from the grammatical, lexical, and prosodic to the stylistic, sociolinguistic and even discursive, as will be shown below. Particular to mouthings as a category of mouth actions is that they reproduce segments of the surrounding spoken language, which is commonly held to be their ultimate origin. This hypothesis will be challenged in the following (see section “New insights into the origin of mouthings”), based on our findings from a corpus-based study of RSL, while we additionally take up the question of which syllables become discarded in reduced mouthings and why. Overall, our findings stand to contribute to the ‘state-of-the-art’ presented here with regard to the form, frequency, functions and origin of mouthings.

### Russian Sign Language

Russian Sign Language is used by Deaf and hard-of-hearing people in Russia and several other formerly Soviet countries. According to the latest census in 2010, 120,000 people in the Russian Federation use this sign language. Although it evidently has a higher number of signers as compared to many other European sign languages, it still remains considerably understudied.

The emergence of RSL is attributed to the foundation of the first Russian school for deaf children in Pavlovsk in 1806. Some researchers believe RSL to be related to French Sign Language (LSF), as the first teachers in the Pavlovsk school had been trained in France and Vienna. This issue, however, remains open to debate. Bickford (2005), who carried out a lexical comparison of East European sign languages, found no evidence in favor of this hypothesis.

Russian Sign Language has been legally recognized as a full-fledged language of the Deaf in Russia and received its official status on December 30, 2012. This means that any time Deaf or hard-of-hearing people contact state, municipal and judicial authorities, they have the right to receive the services of an interpreter trained in RSL. Official recognition of a sign language should help to improve the quality of life and education for the Deaf, though, as the history of sign language development shows, it takes time from the moment of official recognition before real measures of state support become implemented.

Russian Sign Language is unfortunately still largely ignored in the education system for the Deaf in Russia. Deaf people in Russia are primarily taught to write and read standard Russian. In the first decades of the 20th century, the oral method of Deaf education prevailed in Russia. But due to the growth of urban Deaf communities via significant migration flows from villages to cities and the need to provide the Deaf with the basics of knowledge in a short time, numerous evening schools and workshops to eliminate illiteracy (so called *likbezy^4^*) have appeared. Quite obviously, it is impossible to succeed in such tasks without the use of sign language. Therefore, in 1938 at the All-Russian Conference of Deaf Educators, the “purely oral method” was declared unacceptable. After that, both deaf and hearing teachers with knowledge of sign language were allowed to work. However, at the very beginning of the 1950s, there was a major step backwards. In one of his publications, Stalin argued that sign language is not a proper language. And although Joseph Stalin was not an expert in either education or linguistics, this publication was deeply influential, and the purely oral method prevailed again: the deaf were required to learn to speak. Many doctors and educators considered the deaf to be defective, while sign language as a means of interpersonal communication was also regarded extremely negatively – it was banned even outside of school hours at educational institutions. Unfortunately, the echoes of this discriminatory attitude toward the Deaf community and their language is still palpable, and the prejudiced notion that the use of sign language prevents mastery of spoken Russian is still very common among teachers and professionals, as well as among hearing parents in Russia (Zajceva, 2000).

A general scientific interest in RSL arose only in the 1980s (Zajceva, 1987; Grenoble, 1992). Most of the research on the structure of RSL has, so far, been conducted by Zajceva^5^, a distinguished sign language researcher, interpreter and educator, who has studied RSL mostly from a pedagogical perspective (Zajceva, 2000). Selected aspects of RSL grammar have also recently been described (Prozorova and Kibrik, 2007; Kimmelman, 2009, 2012, 2014; Prozorova, 2009; Burkova, 2012, 2015; Burkova and Varinova, 2012; Korol’kova, 2013; Filimonova, 2016; Burkova and Kimmelman, 2019; Kyuseva, 2020). In many respects, RSL appears to be typologically similar to the other urban sign languages described so far. However, RSL is in closer contact with its surrounding spoken and written language, Russian, and is expected to be more deeply affected by it. Studies on mouthings in RSL are, nevertheless, still very scarce (Bauer, 2018, 2019).

## Materials and Methods

This study constitutes the first detailed description of the forms and functions of mouthings in RSL. In particular, we are interested in possible differences in the characteristics of mouthings that are co-articulated with various lexical types of signs, namely the set of core, fully-lexicalized signs, the open class of more spontaneous, partially-lexicalized signs and fingerspelled signs. An adequate description of this sort requires investigating the natural occurrence of the phenomenon in a variety of contexts. To achieve this goal, we conducted a corpus analysis of RSL mouthing. This section explains the methodology of that study. It describes the two corpora that served as our data sources (“The Russian Sign Language Online Corpus” and “The ‘Spot-the-Difference’ Corpus”) and discusses the research questions that will be answered in the remainder of this article (see section “Research Questions”).

### The Russian Sign Language Online Corpus

The Russian Sign Language online corpus^6^ is a currently maintained documentary corpus of RSL and was used as the main data source for our study. The RSL Corpus was built by Svetlana Burkova (Novosibirsk University) and her research group during a documentation project funded by the Russian Foundation for Basic Research (Burkova, 2012, 2015). The corpus currently includes over 230 texts filmed from 43 RSL signers – men and women aged from 18 to 63 years, with varying degrees of deafness: deaf and hard-of-hearing. A large proportion of the signers currently resides in Novosibirsk, a significant number also in Moscow. Since only little research has been done on dialectal variation in RSL, the signing in Novosibirsk and Moscow is not considered to represent different RSL dialects. Burkova and Varinova (2012) have shown that the lexical variation in the signing used in Novosibirsk and Moscow is low and restricted to just some lexical domains (e.g., food and kinship). Moreover, variation occurs only in certain parameters (mostly in movement) and is noticed mainly among younger signers.

The corpus consists of various text-types. It contains spontaneous language production (narratives and dialogues) and texts filmed on the basis of stimulus materials (cartoon retellings, picture-based storytelling). The corpus reflects the true everyday language use of different groups of RSL signers in a variety of situations. While recording the data, in order to maximally exclude the influence of spoken Russian, the signer’s addressee was always a native RSL signer.

For the present analysis of mouthings, 136 video files from 35 native RSL signers were analyzed. Six of the signers were from Moscow and 29 from Novosibirsk. There were 15 women and 20 men. Seven of the signers were hard-of-hearing, one deafblind and 27 deaf. The total duration of the RSL corpus data annotated for the present analysis is 4 hours and 35 minutes. These and other annotations are planned to be made available online.

**FIGURE 1:**
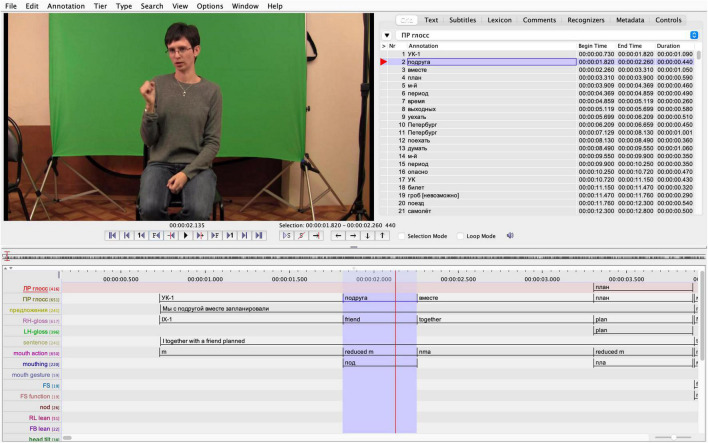
ELAN screenshot. Reproduced with permission from Svetlana Burkova (Novosibirsk State Technical University), available at the online corpus of Russian Sign Language, http://rsl.nstu.ru/.

All mouth activity was carefully examined, and all mouth actions were categorized and annotated using ELAN annotation software (see Figure 1 for a screenshot from this software). We conducted a statistical analysis of this data using R, with a multivariate logistic regression model being used to identify predictors of mouthing and mouth gestures in the data. The RSL corpus is annotated with sign glosses in tiers for the right and left hand, as well as in Russian translation. For the analysis of the mouthings, additional ELAN tiers were added to describe the produced mouth actions. Apart from annotating mouth gestures and cases with no mouth action, the following types^7^ of mouthings were annotated on extra tiers in line with earlier investigations of mouthings in NGT (Bank et al., 2011), for better comparability.

**Table:** 

full mouthing	a manual sign is combined with a complete (i.e. unreduced) mouthing and the whole word is mouthed clearly, e.g., the RSL sign WINDOW is accompanied by the mouthing *okno* ‘window’;
reduced mouthing	a form of mouthing in which only some syllables or even just some elements/sounds of a lexical item are mouthed, e.g., the RSL sign GIRLFRIEND is accompanied by the mouthing *pod(ruga)* ‘sister’; the part of the lexical item in brackets is not visible on the lips (see Figure 1);
standard mouthing	the form of mouthing that occurs most frequently, where the mouthed lexical item and the co-occurring manual sign denote the same semantic concept, e.g. the RSL sign HOUSE is accompanied by the mouthing *dom* ‘house’;
free mouthing	a mouthing without a co-occurring manual sign, e.g., *a* ‘but’ and *nu* ‘well.’
variant mouthing	a form of spoken lexical item that differs from the standard mouthing, e.g. the sign HOUSE is accompanied by the mouthing *domašnjaja* ‘domestic’ and not the standard (most frequently occurring in the corpus) *dom* ‘house’;
inflected mouthing	a form that resembles an inflected Russian lexical item, e.g. *dom-a* ‘house-GEN.SG’ with the sign HOUSE;
overlapping mouthing	a spread mouthing, i.e. when a mouthed form anticipates or extends past the manual production.

The “mouth actions” tier contains 27,377 annotations. At this number, the RSL dataset appears to be larger than the corpora of previous studies on mouthings in Auslan (17,002) or NGT (11,905). The additional “mouthing” tier reflects the exact visible articulation of Russian lexical items or their parts.

All of the annotations of mouth actions were initially made by two annotators: a Deaf native RSL signer also fluent in Russian and a professional sign language interpreter. All annotations were double checked and all cases of doubt^8^ reviewed and discussed with the first author.

For a subset of 2000 randomly chosen signs in the corpus, we added a part-of-speech tier and annotated the belonging of each sign to its respective grammatical class. The tagging was mostly influenced by the semantics of the sign’s use. This way, mouthings can be analyzed in relation to grammatical class for sign glosses.

### The ‘Spot-the-Difference’ Corpus

The ‘Spot-the-difference’ corpus, developed by Kyuseva (2020), served us as an additional source for partially-lexicalized signs. Following Brennan (1992), Johnston and Schembri (1999), and Sutton-Spence and Woll (1999) we define partially-lexicalized (or productive) signs as signs that change their form depending on the context and which form their meaning in a compositional way out of the meanings of their various parts. Partially-lexicalized signs exhibit multiple differences to fully-lexicalized signs (see e.g., Aronoff et al., 2003). However, the extent of these differences has not been studied in detail with respect to mouthing. The current study aims to provide a description of mouthing as it appears in both fully- and partially-lexicalized RSL signs in order to capture potential qualitative differences between the two groups.

Our study focuses on one of the most understudied types of partially-lexicalized signs, namely size and shape specifiers, or SASSes. These are signs that describe the visual characteristics of objects, such as ‘thin,’ ‘thick,’ ‘round,’ ‘angular’ etc. Since these signs do not occur in general conversation often, the RSL online corpus presently does not contain a sufficient quantity of them for our purposes. As a complimentary data source, we used recordings of semi-spontaneous signing collected in the study of RSL SASSes by Kyuseva (2020). These data were collected on the basis of the communicative game ‘Spot-the-difference.’ In this game, two participants are presented with a different version of a cartoon-like picture. They have to collaborate to find 10 differences between the images without looking at each other’s pictures. The stimuli were developed by Kyuseva (2020) to elicit various size and shape descriptions.

This corpus has the same annotational format as the RSL online corpus and includes sentence translations, left- and right-hand glosses and a similar detailed description of mouth actions. For this study, 6 video recordings with a total duration of 28 minutes were used. The signers were four deaf women and two deaf men, native RSL users from the Moscow area. This corpus contributed 598 SASSes to our sample set. It serves as the main data source for the discussion in section “New insights into the functions of mouthings” on the functions of mouthings.

### Research Questions

Based on the corpus data described above, this paper aims to answer the following research questions:

(1) Are mouthings as frequent in RSL as they are in other sign languages? Do they exhibit the same features over all types of signs?
  There is anecdotal evidence from Deaf RSL signers to the effect that RSL uses significantly less mouthing than do some other urban sign languages, such as DGS or NGT. Section “New insights into the distribution patterns of mouthings” of this paper explores this point by analyzing the RSL online corpus. It presents the general frequency of RSL mouthings, as well as their distribution by grammatical classes and by different types of signs.
(2) Do mouthings perform the same functions in RSL as they do in other sign languages that have previously been described?
  The functions of mouthings in sign languages have been explored mainly based on data pertaining to core, fully-lexicalized signs. The current study includes partially-lexicalized signs into the sample set, which has led to the discovery of a new function. Section “New insights into the functions of mouthings” provides a detailed discussion of this issue.
(3) How and why does the reduction of mouthings occur in RSL? Is the stressed segment of the spoken Russian word always mouthed in RSL?

Being surrounded by a spoken language with variable stress and concomitant vowel reduction, RSL is well-suited to contribute to the existing studies on the reduction of mouthings. Section “New insights into the origin of mouthings” presents a statistical analysis of the reduction patterns of RSL mouthings, which results in new insights into the origin of mouthings.

## New Insights Into the Distribution Patterns of Mouthings

An initial analysis of mouthings in RSL on the basis of a set of twenty frequently occurring signs (Bauer, 2019) has indicated that RSL differs from other recently studied sign languages with respect to the proportions of signs found to co-occur with mouthings. Bauer (2019, p. 27) demonstrates that RSL mouthing rates are quite low when compared with Auslan or NGT. In this section, we expand upon the previous description of mouthings in RSL based on a larger dataset (see “The Russian Sign Language Online Corpus” for a description of the analyzed data) and present new insights into mouthing by showing how frequently mouthings occur in RSL, how they are distributed over different parts of speech and sign types and which spreading patterns are most prevalent.

### Frequency of Mouthings

**FIGURE 2:**
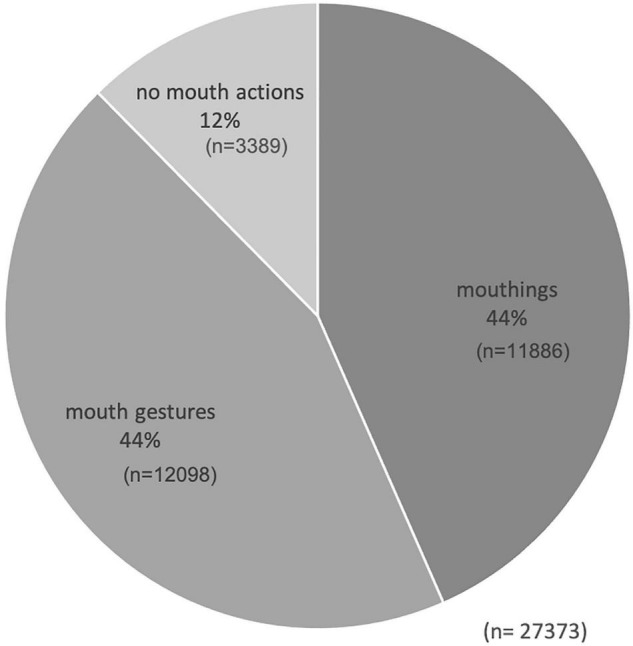
Mouth actions in the RSL corpus.

Our RSL data show that mouthings often accompany manual signs, but they occur far less frequently than has been reported for other sign languages. At the same time, the overall distribution of mouth actions is not unlike that of the other sign languages described to date (see Figure 2). 88% of all manual signs in the RSL corpus are accompanied by some mouth activity, either a mouthing or a mouth gesture. In contrast, the mouthing rate in our RSL data is 44%. This means that only 44% of manual signs were accompanied by mouthings, which is significantly lower than the rates reported by comparable corpus-based studies with several similar text-types, i.e. monologues, dialogues and elicited language production: for NGT, 73% (Bank et al., 2011) and, for Auslan, 56% of all manual signs were reported to co-occur with mouthings (Johnston et al., 2016). Excluding all sign tokens with no mouth action, the mouthing rate becomes 50%, which is still quite low as compared to NGT (80%) or Auslan (73.6%) (Bank et al., 2011; Johnston et al., 2016).

We analyzed the form of RSL mouthings to find out how frequently they are affected by temporal reduction. RSL mouthings came in a reduced form in 75% (*n* = 8904) of all cases (*n* = 11886). This finding is also quite surprising, because an opposite tendency has been reported for Auslan and STS, where fully-articulated mouthings were found to be the most common category (Johnston et al., 2016; Mesch and Schönström, 2021). In RSL, full mouthings are generally short, being no longer than two or three syllables. Exceptions to this are mouthings that accompany fingerspelled signs. Those that co-occur with fingerspelling are always fully articulated in RSL.

**FIGURE 3:**
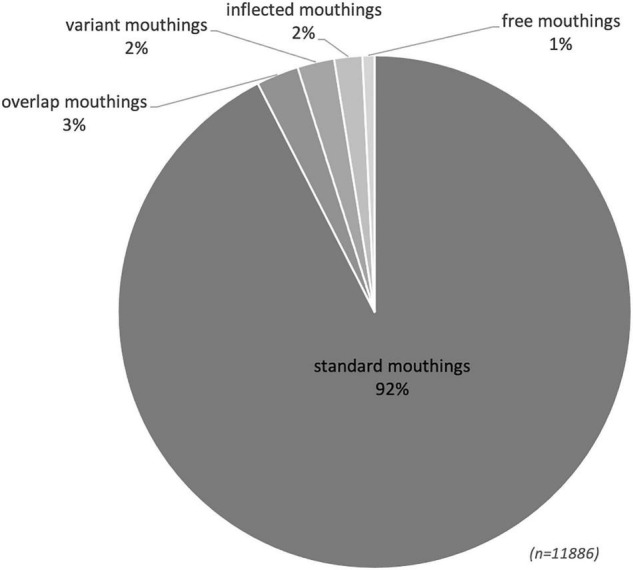
Types of RSL mouthing.

Figure 3 shows that the overwhelming majority of signs pair with a standard mouthing. 92% of all mouthings in the RSL corpus denote the same semantic concept as their co-occurring manual sign. In 3% of all cases, the mouthing was spread regressively to the previous manual sign or progressively to the following manual sign. Cases of variant mouthing^9^, i.e., when the form of the spoken lexical item differs from the standard mouthing, were quite infrequent. Inflected mouthings occurred in only 2% of all cases. These were mostly inflected for case (e.g. *škol-u* ‘school-ACC.SG’ together with the sign SCHOOL). 1% of all mouthings in the RSL data were free mouthings. These are isolated words or even short phrases that occur without an accompanying manual sign, or while the hands are resting. Referred to as solo mouthings elsewhere (Bank, 2014), they are often used as a backchannel, i.e., as a short feedback cue, e.g., when a signer mouths *da* ‘yes.’ Further examples of mouthings without accompanying manual signs are *tože* ‘also,’ *a* ‘but’ and *nu* ‘well.’

### Distribution Over Grammatical Class and Sign Type

**FIGURE 4:**
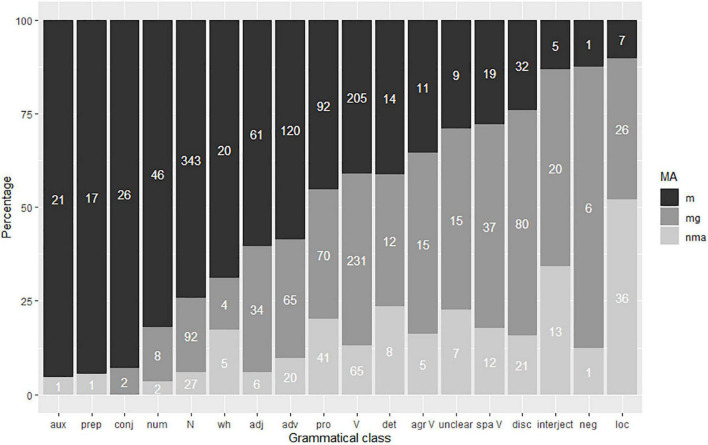
Mouth action rates by grammatical class (ranked by decreasing % of mouthing).

The grammatical class of a sign is known to be a significant factor in predicting the co-occurrence of a mouthing (Kimmelman, 2009; Johnston et al., 2016). Some grammatical classes, such as nouns, prepositions and conjunctions, favor the use of mouthings, while others, such as verbs or pronouns, disfavor their use. Our study partly replicated the design of Johnston et al. (2016), in which the distribution of Auslan mouthings was investigated over signs of various grammatical classes. Our results confirm earlier findings. Mouthing rates in RSL vary significantly according to the grammatical class of the accompanying manual sign (see Figure 4 and Supplementary Table 1). Based on RSL corpus data, mouthings co-occur with nouns more often than with verbs. Apart from with nouns, the highest mouthing rates in the RSL corpus were with function words (auxiliaries, prepositions, conjunctions and *wh*-question words) and numbers. Spatial verbs, discourse markers, interjections, negators and locatives most strongly disfavored the use of mouthings (see Figure 4). This distribution of mouthings over signs of different grammatical classes is comparable to that of other sign languages.

Not only the grammatical class of a sign but also its type appears to be a significant predictor of the use of mouthing. The study of Auslan has already shown that different sign types (e.g., core lexicon, productive lexicon, and gestural elements) exhibit very different rates of coarticulated mouth actions (Johnston et al., 2016). This is also true for RSL. The presence of mouthings varies in accordance with the type of the sign that it accompanies.

**FIGURE 5:**
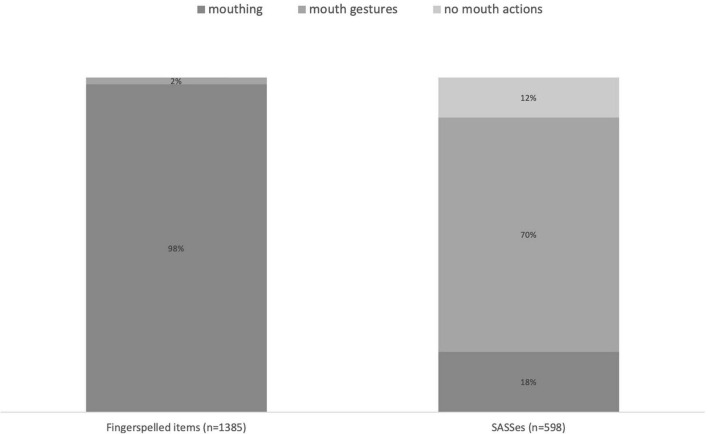
Fingerspelling, SASSes, and mouthings.

To demonstrate this, let us contrast two sign types which differ extremely in their rate of co-occurrence with mouthings: fingerspelled items^10^ and SASSes (see Figure 5). Similar to findings on Auslan, fingerspelling most strongly correlates with the use of mouthings. In the corpus data, RSL signers mouthed 98% of their fingerspelled items. Occasionally, a particular type of fingerspelled elements, loan signs, occurred without mouthings, which accounts for the remaining 2% of cases. The mouthings that accompany fingerspellings appear to obligatorily be standard mouthings, which are fully articulated.

By contrast, only 18% of SASSes in the corpus were accompanied by a mouthing (see Figure 5). Interestingly, although SASSes prototypically denote physical characteristics, the mouthings that do accompany them never represented Russian adjectives of size or shape. Instead, they were silent articulations of Russian nouns for concrete objects (e.g. *doska* ‘plank,’ *mjač* ‘ball’ or *rama* ‘frame’). The resulting sign functions as a noun that denotes an object of a particular shape, as seen in example (4).


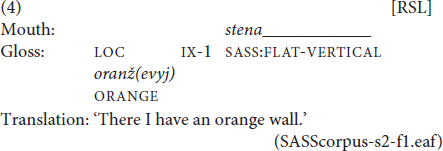


In (4), the SASS describing a flat vertical object is accompanied by the mouthing *stena* ‘wall’ and thereby denotes a flat vertical wall. In section “New insights into the functions of mouthings,” we argue that mouthings in examples like this signal that the SASS has lost its compositional semantics and should be interpreted as a fully-lexicalized sign.

In contrast to their behavior in the case of fingerspelled signs, the mouthings that accompany SASSes frequently exhibit reduction. Full mouthings appeared only when the Russian word in question had no more than two syllables (as in *doska* ‘plank,’ *krug* ‘circle,’ *stupen’* ‘step’). Most of the mouthings accompanying SASSes are standard in RSL (in terms of their semantic congruence with the manual sign). Variation was encountered only in two tokens, namely the variants *dom* ‘house’ versus *budka* ‘cabin’ accompanying a SASS denoting a three-dimensional object with a pointed end, and the variants *doroga* ‘road’ versus *tropa* ‘small path’ accompanying a SASS denoting a long narrow object.

### Spreading Patterns

For a subset of eight randomly chosen files from the RSL corpus (3406 tokens), we counted all instances of spreading. Overall, we observed 52 spreading. Out of them, 41 mouthings were spread from the source sign over just one other adjacent sign, as in (5), three instances saw the mouthing spread from the source sign over several sequentially adjacent signs, as in (6), and eight instances were spreading of a mouthing without a manual source over an adjacent sign, as shown in (7).


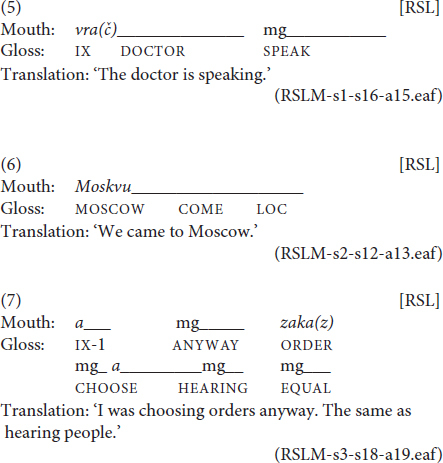


In example (5), the mouthing of *vrač* ‘doctor’ is spread from its source over an adjacent pointing sign preceding it. In (6), the mouthing of *Moskvu* ‘Moscow.ACC’ is spread from its source sign over two subsequent signs, namely to the classifier predicate ‘to come’ and a locative marker with the meaning ‘there.’ Finally, in example (7), the second instance of *a* ‘but, and’ is a free mouthing which does not have a manual counterpart. This mouthing is spread over two signs, CHOOSE and HEARING, on the boundary of two phrases. This latter type of spreading is not often discussed in the literature. However, our data suggest that it has a function similar to some of the more “typical” spreading examples and should therefore be included in the sample set.

Out of 44 instances where a mouthing was spread from a co-occurring manual sign, 31 were progressive, 12 were regressive and 1 was mixed, in that it exhibits spreading over two additional signs, both before and after the source sign. The free use of regressive spreading sets RSL apart from such languages as NGT and BSL and brings it closer to SSL, which also exhibits occasional use of regressive spreading (Crasborn et al., 2008; Mesch and Schönström, 2021). Tables 1.1 and 1.2 show the source and target signs of spreading in order of their frequency.

**TABLE 1.1:** Source signs of spreading.

Part of speech	Tokens
Noun	15
Verb	11
Adverb	6
Conjunction	6
Adjective	3
Numeral	1
Pronoun	1
WH-word	1

**TABLE 1.2:** Target signs of spreading.

Part of speech	Tokens
Pointing sign	19
Verb	6
Noun	6
Palms up	4
Classifier predicate	3
Adjective	3
Fingerspelled word	1
Negative marker	1

Previous research on sign languages has indicated that mouthings spread for the most part from content signs to functional signs (Sandler, 1999; Boyes-Braem, 2001; Crasborn et al., 2008; Bank, 2014). To an extent, our data confirm these observations: the most frequent sources of spreading were nouns, verbs and adverbs; and the most frequent target of spreading was a pointing sign. However, contrary to the stated generalization, mouthing can also spread to a content word, such as a verb (six instances), a noun (six instances) or an adjective (three instances). Strikingly, it can even spread from a functional sign, such as a conjunction (six instances) or a *wh*-word (one instance). We argue that these mismatches in the direction of spreading are connected to the function that the spreading performs in the sentence. We will now turn to the functions of spreading attested in our data.

As we discussed in section “Mouthing Functions,” the spreading of a mouthing is considered to be one of the markers of phonological constituents in sign languages. In other words, it contributes to breaking a series of articulated signs into patterns of rhythmic and intonational structure. Some of the phonological phrases formed by the spreading of mouthings are isomorphic to syntactic structures. Indeed, in our data, mouthing binds such elements as compounds (COUNTRYSIDE HOUSE; mouthing *dača* ‘country-house’), noun phrases [FIRST DAY; mouthing *perv(yj)* ‘first’], verb phrases [CANNOT APPLY; mouthing *mo(žet)* ‘can’] and predicates with their subjects [IX-1 REFUSE ‘I refused’; mouthing *otkaza(las’)* ‘refused.F’]. These spreading, for the most part, have a progressive direction.

The observed type of prosodic binding that does not conform to the syntactic structure of the sentence is represented exclusively by spreading to pointing signs. These spreading can be progressive or regressive; see (8–9).


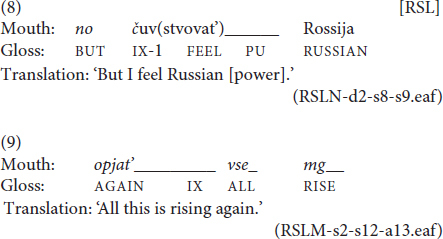


In (8), the mouthing of *čuv(stvovat’)* ‘to feel’ binds the head of the verb phrase without the argument and the subject. Since the subject, which is represented by a pointing sign, occurs prior to the verb, the spreading has a regressive direction. In (9), the mouthing of *opjat’* ‘again’ progressively binds the index subject and the adjunct of the predicate.

In 19 examples from our sample set, the spreading not only bound signs from a single phrase, but rather connected two clauses. In (10–11), below, the mouthing starts at the last sign of the first phrase and is spread over the first sign of the second phrase:


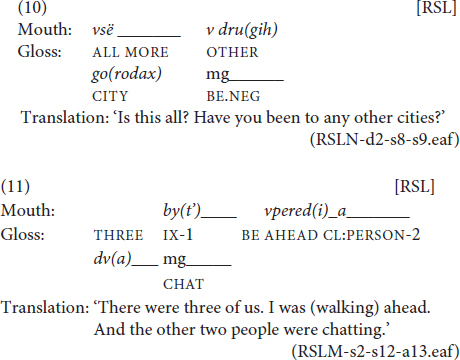


In (10), the mouthing of *vsë* ‘all’ starts at the only manual sign of the first phrase, ALL ‘Is this all?,’ and is spread from there over the first sign of the second phrase, MORE. In (11), the mouthing of *a* ‘but, and’ starts at the last sign, AHEAD, of the phrase ‘I was (walking) ahead’ and is spread over the first sign, CL:PERSON-2 (a classifier predicate denoting two people), of the following phrase ‘… the other two people were chatting.’ This example [as well as example (7), above] shows the spreading of a mouthing that does not have a designated manual source. Note that both signs, AHEAD and CL:PERSON-2, have their own semantically congruent mouthings which accompany them. The mouthing *a* ‘but, and’ finds a place between them, connecting the end of the sign AHEAD and the beginning of the sign CL:PERSON-2.

The mouthing of *a* is a silent articulation of the Russian conjunction *a*, which can denote coordination (the meaning ‘and’) or contrast (the meaning ‘but’). When used at the beginning of a sentence, the Russian word *a* can perform the discursive function of the connective, ensuring the coherence of the narrative and marking the continuation of the speaker’s turn. In this sense, it is functionally close to the English markers *and, but* and *or* (Schiffrin, 1987; Chafe, 1994; Fraser, 1996). Examples like (11) allow us to put forward a preliminary hypothesis that RSL has borrowed this strategy of connecting utterances. In order to signal that the turn of the speaker is still not over, a signer can mouth the conjunction *a* ‘but, and’ over the sentence boundary, or just spread the mouthing from the last sign of the first phrase to the first sign of the next phrase, as in example (10). Only in this function is the spreading of a mouthing that does not have a designated source sign possible. Moreover, in this function, a mouthing can spread from a functional sign to a content sign. In our sample set, we attested multiple instances of mouthings spreading regressively from the conjunction BUT (the first sign of a following sentence) to verbs, adverbs and nouns.

The crucial difference between the use of such markers in spoken Russian and the RSL strategy lies in their corresponding frequencies. While Russian speakers use connectives such as *a* ‘and, but,’ *i* ‘and’ or *no* ‘but’ on a regular basis, RSL users do not seem to do so very frequently. It is possible that the spreading of a mouthing as a discursive connective has a secondary status to such sign language internal markers as, for example, the weak hand hold (see Kimmelman, 2014). More data is needed in order to confirm the function of this type of spreading and to establish its role in RSL discourse.

### Summary

To sum up the findings of this section, we have seen that mouthings in RSL are relatively infrequent (with a 44% occurrence rate) when compared to other sign languages for which comparable corpora exist. We interpret this as an indication of cross-linguistic variation in the domain of mouthing. Another interesting finding is the prevalence of reduced mouthings in the RSL corpus (75% of all instances).

Emulation of the Auslan study enabled a cross-linguistic comparison of the distribution of mouthings in RSL over signs of different grammatical classes. Our RSL data in many ways confirm earlier findings on the occurrences of mouthing in relation to grammatical class. To demonstrate that different sign types also exhibit very diverse rates of co-articulation with mouth actions, we contrasted the frequency of their occurrence in tandem with fingerspelled elements versus with SASSes. While fingerspelled signs strongly favored co-articulation of mouthings, SASSes were only very seldom accompanied by them.

With regard to spreading patterns, we found that the most common type observable for mouthings in RSL is the progressive one, but mouthings were also observed to be spread regressively. Moreover, we showed that mouthings are spread, for the most part, from content signs to functional signs. However, we also attested instances of spreading in the opposite direction, which can be connected to a specific function that this spreading performs in the discourse as a means of indicating that one’s turn is not yet over.

## New Insights Into the Functions of Mouthings

The previous section described various aspects of the form of mouthings in RSL and placed them in a typological context. This section focuses on the functions that mouthings perform in RSL. It firstly enumerates the functions that have already been established in the data from other sign languages and then discusses an additional function that has not been attested before, namely disambiguation between different sign types.

### Mouthing and Degrees of Lexicalization

**FIGURE 6:**
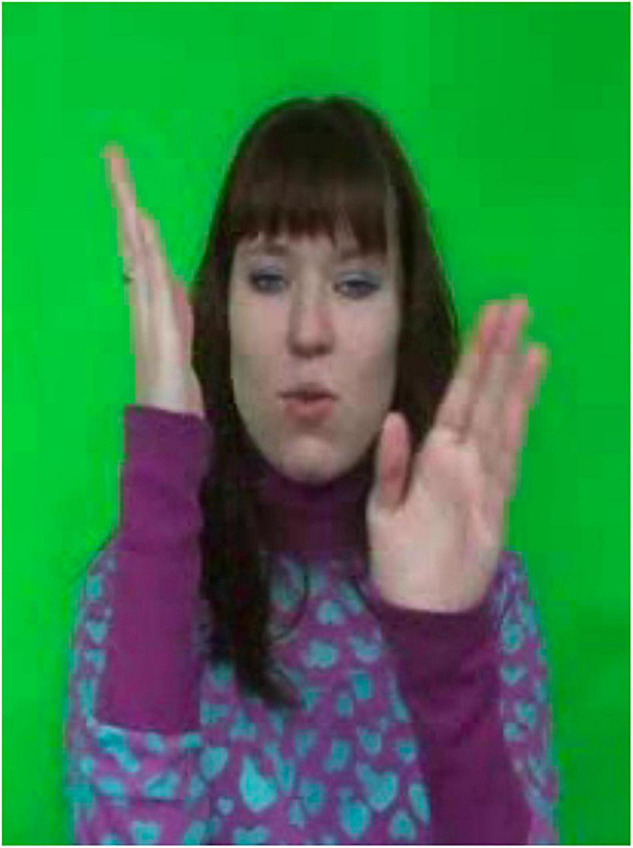
RSL sign weather. Reproduced with permission from Svetlana Burkova (Novosibirsk State Technical University), available at the online corpus of Russian Sign Language, http://rsl.nstu.ru/.

Russian Sign Language mouthings serve a wide range of functions, most of which have already been attested in other sign languages. These are the phonological function, the prosodic function and the grammatical function. The phonological function, i.e. lexical disambiguation, can be illustrated by means of the RSL sign in Figure 6, below. The manual part of this sign involves two vertical flat hands that simultaneously move towards and away from each other several times.

Depending on the accompanying mouthing, this sign can have the meanings: ‘weather’ (with a mouthing of *pogoda*), ‘climate’ (mouthing: *klimat*) or ‘fate’ (mouthing: *sud’ba*). While the first two meanings are connected by a metonymic relationship (a temporal versus a permanent property) and can be interpreted as two meanings of a polysemous sign, the third one does not have an obvious semantic relationship to the former two and therefore represents a clear case of a different lexeme.

The prosodic function of syntactic binding was discussed in Section “Spreading Patterns” on spreading patterns, above. Examples (5–9) showed how the spreading of mouthings contributes to breaking up a chain of signs into rhythmically and intonationally coherent chunks. Finally, the grammatical function could be seen in the distribution patterns of mouthings with different parts of speech (Figure 4). Similar to in Auslan and NGT, mouthings in RSL have a tendency to co-occur with nouns and adjectives. This is in line with the finding of Kimmelman (2009) that, in RSL, mouthings constitute one of the phonological mechanisms that help to distinguish between nominal and verbal signs.

The inclusion of partially-lexicalized manual signs in the sample set allowed us to discover a new function that mouthings can have in a language, namely that of disambiguating between signs of different types. Our data on the co-occurrence of mouthings with partially-lexicalized signs come from the ‘Spot-the-difference’ corpus, described in Section “Research Questions,” above. This corpus provided 598 instances of SASSes to the sample set. It is important to understand, however, that, with respect to sign type, SASSes do not represent a homogeneous group in RSL. Rather, they can occupy different positions on the “lexical continuum”. In employing the notion of this continuum, we follow usage-based approaches to sign language linguistics, according to which signs (or parts of signs) can be at various degrees of entrenchment into a speaker’s linguistic knowledge (Lepic, 2019). We see fully-lexicalized and partially-lexicalized signs as extremes of the lexical continuum and we acknowledge the existence of signs that represent intermediate cases.

**FIGURE 7:**
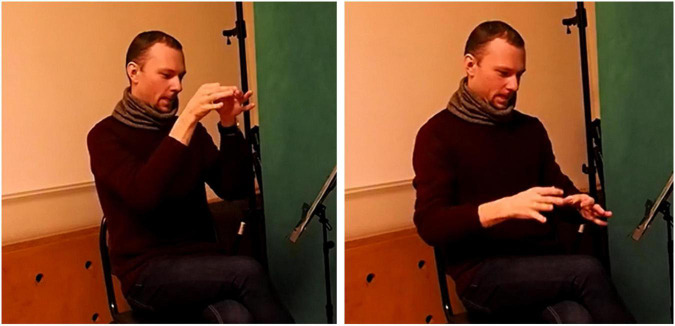
SASS:tall-vertical-conical (about a vase).

Irrespective of their position on the continuum, the manual components of SASSes iconically depict the visual characteristics of objects. The difference between more- versus less-lexicalized SASSes lies in the nature of the meaning they express. While the meaning of prototypical partially-lexicalized SASSes is compositionally formed out of the meanings of their sub-sign elements, more-lexicalized SASSes have non-compositional semantics. Figure 7 represents a typical partially-lexicalized SASS.

This sign appears in Example (12) and describes a tall conical vase. The meaning of this sign is formed by combining the meanings of its components: the closed handshapes at the beginning of sign production indicate a narrow hole on top of the vase, the open handshapes at the end of the sign show its wide bottom; the downward movement of the hands signals the vertical orientation of the object; the size of the movement indicates that the vase is tall; and the trajectory depicts the straight smooth shape of the vase’s sides.


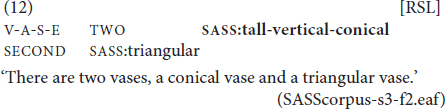


**FIGURE 8:**
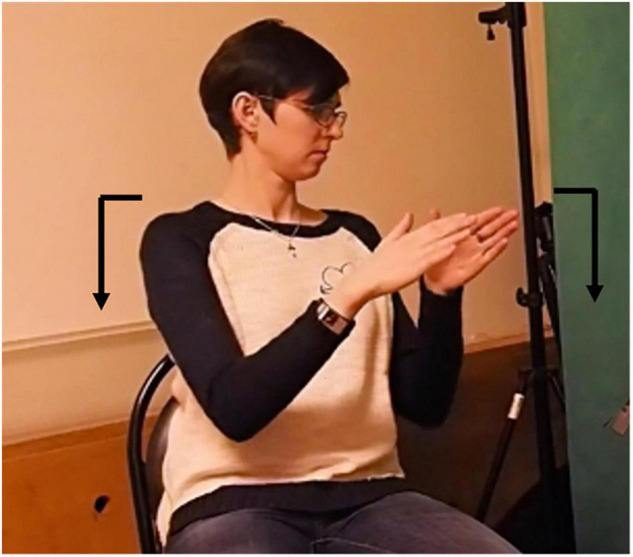
Lexicalized SASS: chest of drawers.

Figure 8, below, gives an example of a SASS that is located closer to the fully-lexicalized end of the continuum. This sign appears in (13) and denotes a chest of drawers. In the same way as in the previous example, the manual components of the sign describe various aspects of its visual characteristics, i.e. the flat hands denote the wide surfaces, the angular trajectory shows the shape of the object etc. However, the sign does not denote just any three-dimensional cubical object, but specifically a chest of drawers. This meaning cannot be arrived at from the elements of the sign, it is a non-compositional part of the semantics.


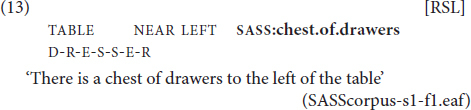


We used the presence of non-compositional semantics as the main criterion for determining the status of the sign, i.e., whether it is more- or less-lexicalized. The two groups were unequal in size, with the former comprising 197 elements and the latter – 401 elements. Each sign was marked according to the type of mouth articulation that accompanied the manual part. The three options were: mouthing, mouth gesture and no mouth activity. The bar chart below shows the distribution of these options in the two groups of signs.

**FIGURE 9:**
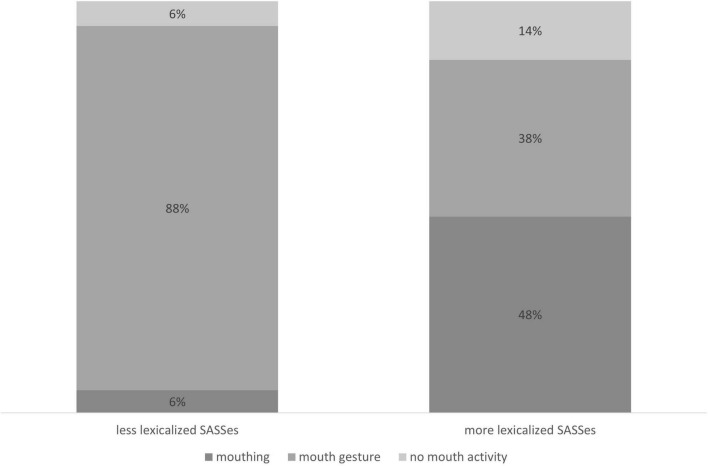
Distribution of non-manual activity types in less-lexicalized versus more-lexicalized SASSes.

Figure 9 illustrates that the three types of non-manual activity are not evenly distributed across the two groups. While the absence of mouth action has a similar percentage in less-lexicalized versus more-lexicalized SASSes, the same cannot be said about mouthing and mouth gestures. Less-lexicalized SASSes exhibit a strong tendency to co-occur with mouth gestures. More-lexicalized SASSes do not show this tendency. Instead, they exhibit a predominance of mouthings. This observation is confirmed by a logistic regression model: the predicted probability of observing mouthing was 0.48 for more-lexicalized SASSes and only 0.05 for less-lexicalized SASSes (logit difference –2.76, *SE* = 0.26, *z* = –10.5, *p* < 0.0001).

Our data contain examples where the same manual sign was accompanied by a mouthing in one instance and by a mouth gesture in another. In the first case, it acts as a noun that denotes a concrete object and, in the second case, as an adjective that describes visual characteristics. For example, if two hands in the small-C shape are moving in opposite directions and are accompanied by a mouthing of *doska* ‘plank,’ then the sign represents the noun PLANK, which is located closer to the fully-lexicalized end of the continuum. If the same manual sign is accompanied by the mouth gesture /u/, then it represents a SASS with the meaning ‘long, thin, narrow’ and is located closer to the partially-lexicalized end of the continuum.

We interpret the function of mouthings in these examples as a newly discovered type of sign disambiguation. Prototypical examples of lexical disambiguation include a fully-lexicalized sign that can be accompanied by different mouthings, depending on the intended meaning (Boyes-Braem, 2001). In our case, the same manual sign can be accompanied either by a mouthing or by a mouth gesture and, consequently, receives either a fully-lexicalized or a partially-lexicalized interpretation. Moreover, the statistically confirmed tendency of mouthings to co-occur more often with more-lexicalized SASSes than with less-lexicalized ones allows us to hypothesize that mouthings represent one of the general phonological mechanisms for distinguishing between the two sign types.^11^ Other mechanisms, discussed in Kyuseva (2019), include the presence versus absence of movement and the syllabic structure.

### Summary

This section described the functions that mouthings in RSL perform in a sentence. We attested the following functions: (1) lexical disambiguation, (2) prosodic binding, (3) a mechanism that helps to distinguish between nominal and verbal signs, and (4) disambiguation between different sign types. The first three functions have been established for such languages as Auslan (Johnston and Schembri, 2007), Israeli Sign Language (Sandler, 1999), NGT (Crasborn et al., 2008), and several others. The last function has not previously been discussed in the literature.

## New Insights Into the Origin of Mouthings

This section is concerned with the most frequent type of mouthing found in the RSL corpus, namely reduced mouthing. We answer here our third question as to how and why the reduction of mouthings occurs in RSL. The findings of this research have led us to re-think and re-hypothesize the source of the mouthing phenomenon.

Linguists have never been in any doubt as to the origin of mouthings. They have always been understood as a spoken-language-based contact phenomenon (Boyes-Braem, 2001; Crasborn et al., 2008; Johnston et al., 2016). Thus, Bank et al. (2011, p. 250) believe that casual spoken Dutch is an important source of NGT mouthings. Nadolske and Rosenstock (2008) described ASL mouthings as being “derived from” or “influenced by spoken English,” and Mesch and Schönström (2021) consider STS mouthings to be “borrowed from the spoken Swedish language.” A recent study has defined mouthings as a “vocal production always borrowed from the surrounding spoken language, subvocalized or almost inaudible, and usually an approximation of the spoken word” (Bogliotti and Isel, 2021, p. 2).

In contrast, we propose in this section, based on the RSL data, that mouthing is not only a spoken-, but also a written-language-based contact phenomenon. Our suggestion is that the written modality may be a primary source for the occurrence of mouthings in RSL and possibly also in some other sign languages. Our study of the reduction patterns of mouthings in RSL (see section “Study of Reduced Mouthings”), as well as our analysis of the visual phonetic characteristics of vowel quality in RSL mouthings (see section “How Mouthings Differ From Spoken Russian Pronunciation”), have led us to conclude that mouthings in RSL are primarily based not on contact with the spoken modality (Russian speech), but rather on contact with the written modality (Russian orthography). In the following, we present the arguments supporting our claim.

### Study of Reduced Mouthings

Reduced mouthings appear to be used quite differently in various sign languages, over various sign types and by various signers. In Auslan, for example, reduced mouthings are quite rare, according to Johnston et al. (2016, p. 21). In RSL, they are overwhelmingly frequent, based on our corpus data: 75% of all mouthings in the RSL corpus occur in their reduced form.

In contrast to DGS, where reduced mouthings occur more often with verbs than with nouns (Ebbinghaus and Heßmann, 1995), in our RSL data, 55% of all reduced mouthings occurred with nouns and only 34% with verbs. Our detailed analysis of 2000 randomly sampled RSL signs in the corpus was unable to confirm a tendency similar to the finding of Ebbinghaus and Heßmann (1995).

Two views exist in the literature as to why only parts of the spoken word are mouthed in sign language. The first hypothesis states that reduced mouthing conforms to the rhythm of the (mono)syllabic form of the manual sign (Sandler and Lillo-Martin, 2006, p. 105). The second hypothesis states that it is the stressed part of the spoken-language word that is usually mouthed, which indicates that signers are familiar with the rhythmic structure of spoken words (Bank, 2014, pp. 40–42). In the NGT corpus data, Bank (2014, p. 40) observed that the reduction of mouthings only affected unstressed syllables such that the stressed syllable of the corresponding spoken Dutch word always remained visible. Similarly, the findings from the Auslan and DGS data showed that reduction in mouthings typically happened in the form of deleting word-final consonants and syllables with a schwa, which are usually unstressed in Germanic languages (Ebbinghaus and Heßmann, 1995; Johnston et al., 2016). The two hypotheses are, of course, not mutually exclusive: a mouthing can conform to the rhythm of the often monosyllabic form of its corresponding manual sign and, at the same time, be reduced to the stressed syllable of a surrounding spoken language word.

Our corpus observations reveal that mouthings in RSL do not necessarily occur in conformity with either of these hypotheses. Consider, for example, the RSL sign HELP. This sign is usually disyllabic (e.g. the number of sequential movements in its form is two in 73% of cases within the RSL corpus) and, in our data, this sign is never accompanied by a mouthing containing the stressed syllable of the spoken Russian word. It is rather accompanied by the first, unstressed syllable of the Russian word *pom*(*ogat’*) ‘to help’ or by the first two unstressed syllables *pomo*(*gat’*) (Table 2).

**TABLE 2:** Thirty RSL signs showing their syllabic structure and the form of the co-occurring mouthings.

RSL gloss (number of tokens)	Russian citation form	Monosyllabic sign (single movement)	Disyllabic sign (double movement)	Full mouthing	1st part of the word [1st syllable or 1st letter(s)]	1st-2nd syllable	2nd syllable	2nd-3rd syllable	3rd syllable	1st-3rd syllable
GRANDMOTHER (*n* = 41)	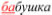 *babuška*	63%	28%	10%	59%	13%	0%	0%	0%	0%
SPEAK (*n* = 21)	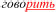 *govorit’*	57%	30%	5%	48%	28%	14%	0%	5%	0%
GIRL (*n* = 64)	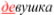 *devuška*	61%	36%	0%	74%	23%	3%	0%	0%	0%
WOOD (*n* = 19)	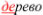 *derevo*	71%	29%	0%	60%	40%	0%	0%	0%	0%
INTERESTING (*n* = 38)	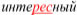 *interesnyj*	76%	24%	0%	39%	33%	0%	0%	0%	28%
COMPUTER (*n* = 22)	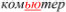 *komp’juter*	43%	57%	0%	72%	28%	0%	0%	0%	0%
BEAUTIFUL (*n* = 19)	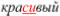 *krasivyj*	95%	5%	0%	50%	28%	5%	11%	6%	0%
STORE (*n* = 22)	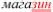 *magazin*	77%	18%	0%	73%	27%	0%	0%	0%	0%
SMALL (*n* = 63)	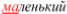 *malen’kij*	95%	5%	0%	85%	5%	10%	0%	0%	0%
MAN (*n* = 46)	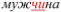 *mužčina*	57%	37%	0%	92%	4%	0%	4%	0%	0%
FOR-EXAMPLE (*n* = 93)	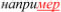 *naprimer*	33%	65%	0%	78%	5%	11%	4%	2%	0%
NOVOSIBIRSK (*n* = 22)	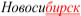 *Novosibirsk*	23%	77%	0%	63%	17%	12%	0%	0%	0%
NORMAL (*n* = 22)	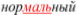 *normal’nyj*	59%	23%	0%	59%	41%	0%	0%	0%	0%
MONKEY (*n* = 21)	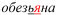 *obez’jana*	33%	67%	0%	80%	20%	0%	0%	0%	0%
RETURN (*n* = 39)	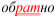 *obratno*	82%	18%	0%	64%	31%	5%	0%	0%	0%
COMMUNICATE (*n* = 25)	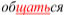 *obščat’sja*	44%	39%	14%	76%	10%	0%	0%	0%	0%
CLASSMATE (*n* = 12)	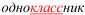 *odnoklassnik*	75%	25%	0%	100%	0%	0%	0%	0%	0%
TENT (*n* = 24)	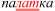 *palatka*	100%	0%	0%	67%	33%	0%	0%	0%	0%
HELP (*n* = 33)	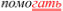 *pomogat’*	21%	73%	0%	64%	36%	0%	0%	0%	0%
CORRECT (*n* = 23)	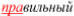 *pravil’nyj*	100%	0%	0%	100%	0%	0%	0%	0%	0%
WORK (*n* = 41)	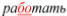 *rabotat’*	42%	51%	0%	77%	15%	0%	0%	0%	0%
CHEER (*n* = 24)	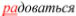 *radovat’sja*	46%	42%	0%	40%	27%	7%	0%	13%	13%
CHILD (*n* = 30)	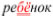 *rebënok*	53%	42%	0%	45%	40%	5%	10%	0%	0%
DOG (*n* = 48)	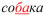 *sobaka*	38%	58%	15%	38%	32%	15%	0%	0%	0%
CALM (*n* = 18)	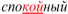 *spokojnyj*	100%	0%	0%	88%	12%	0%	0%	0%	0%
TRY (*n* = 17)	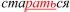 *starat’sja*	76%	24%	6%	41%	29%	18%	0%	6%	0%
COLD (*n* = 17)	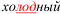 *xolodnyj*	23%	53%	0%	100%	0%	0%	0%	0%	0%
GOOD (*n* = 42)	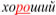 *xorošij*	86%	12%	9%	19%	24%	26%	11%	2%	0%
PERSON (*n* = 20)	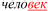 *čelovek*	100%	0%	41%	59%	0%	0%	0%	0%	0%
FEEL (*n* = 35)	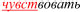 *čuvstvovat’*	57%	43%	0%	94%	6%	0%	0%	0%	0%

*Underlined syllables (marked red) in the second column are stressed in spoken Russian.*

To follow up on this observation, we tested the two stated hypotheses. We use novel data by looking at reduced mouthings in RSL and ask which of the two hypotheses holds true. We thereby posed the following questions concerning the structure and contents of RSL mouthings:

*(1) Do reduced mouthings conform to the rhythm of the (mono)syllabic form of the sign in RSL?*
*(2) Do reduced mouthings contain the stressed syllable of the equivalent spoken Russian word?*

We analyzed 30 signs^12^ in the RSL corpus, as listed in Table 2. The Russian translations of all these signs contain at least three syllables in their citation forms. Overall, we investigated 1400 tokens in detail with regard to the number of their sign movements and the form of the mouth actions they co-occur with in the corpus. The majority of these sign tokens (941) were accompanied by mouthings.

In testing the first hypothesis, we investigated whether the syllabic structure of a manual sign influenced the reduction pattern of an accompanying mouthing. According to the hypothesis, monosyllabic signs are usually accompanied by monosyllabic mouthings, and disyllabic signs should co-occur with disyllabic mouthings. Following Brentari (2019), we define sign language syllables in terms of the number of sequential movements in a sign’s form. Reduplication of a sign’s form thus generates another syllable. The difficulty in testing this hypothesis consists in obtaining an adequate number of disyllabic signs in the data. As is well known, sign languages exhibit a tendency toward a monosyllabic sign structure. In ASL, more than 80% of forms are monosyllabic, and only 17% are disyllabic (cf. Stokoe et al., 1965; Brentari, 1998). There has been no similar investigation of syllabic structure in RSL, but we were able to confirm the monosyllabic tendency through our corpus-based observations. Out of the 30 selected RSL signs (see Table 2), we interpret only eight signs as being disyllabic, as they were produced with a double movement in more than 50% of the cases in our corpus data. The majority of the signs in our sample set are monosyllabic (21). In Table 2, below, the monosyllabic signs (in the third column) are marked by a light grey color and the disyllabic signs (in the fourth column) by a dark grey color. One sign (CHEER, *n* = 24) may be considered mono- and disyllabic because it occurred monosyllabically in 46% of cases and disyllabically in 42% of cases within the corpus.

**FIGURE 10:**
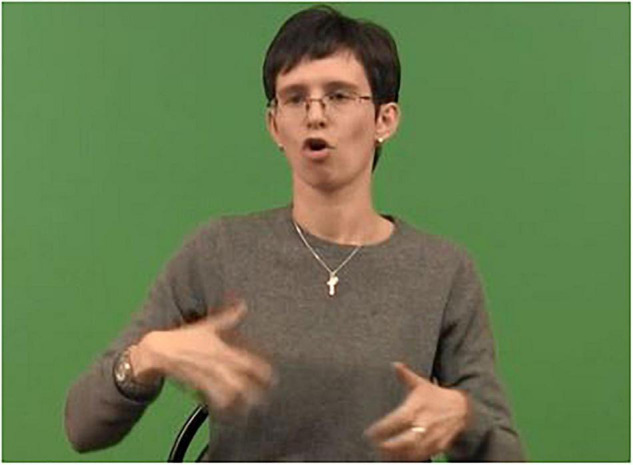
RSL sign monkey. Reproduced with permission from Svetlana Burkova (Novosibirsk State Technical University), available at the online corpus of Russian Sign Language, http://rsl.nstu.ru/.

A glance at Table 2 reveals that not all 21 monosyllabic signs were accompanied by a monosyllabic mouthing in all cases. Some monosyllabic signs do not co-occur with monosyllabic mouthings in the majority of their cases (e.g. more than 50% of all cases). Consider, as an example, the RSL sign INTERESTING. It is a monosyllabic sign (in more than 50% of all tokens) that was accompanied by a monosyllabic mouthing in only 39% of all cases, and by a disyllabic mouthing in 33% and a trisyllabic mouthing in 28% of cases. While monosyllabic signs did tend to be accompanied by a monosyllabic mouthing, disyllabic signs did not occur with a disyllabic mouthing in the majority of cases. Consider the disyllabic RSL sign NOVOSIBIRSK. It was accompanied by a monosyllabic mouthing of *nov* in 63% of all cases. Similarly, the disyllabic RSL sign MONKEY (see Figure 10) co-occurred with a monosyllabic mouthing of *ob* in 80% of all cases in the corpus.

Considering the data in Table 2, we can formulate the following hypothesis to test if there is a correlation between the syllable structure of signs (e.g. their number of movements) and the form of accompanying reduced mouthings (e.g. the number of their visible syllables).

*H1: If a manual sign is disyllabic, its accompanying mouthing is also disyllabic.*
*If a manual sign is monosyllabic, its accompanying mouthing is also monosyllabic.*

For this analysis, we calculated the percentage of all sign tokens in which only one syllable was mouthed (mouthed_1) and that of tokens in which more than one syllable was mouthed (including the few cases of full mouthing) (mouthed_2+). In order to quantitatively assess whether the proportion of mouthed syllables (i.e. one vs. two or more) allows us to draw any conclusions about the syllabic structure of the sign (namely the number of its constitutive movements), we used linear mixed models (performed with Anova) in the R software package (R Core Team, 2016). This entailed fitting two simple linear regression models, which tested, for each mouthing, whether the number of mouthed syllables was a predictor of the syllabic structure of its co-occurring manual sign. In both cases, the two predictors (mouthed_1 and mouthed_2+; see Supplementary Table 1) were not significant, i.e. the proportion of one vs. two or more mouthed syllables was not a significant predictor of the number of movements constituting the manual sign. Thus, we could not confirm a link between the syllabic structure of a given sign and the number of syllables visible in co-occurring mouthings though our statistical analysis of the RSL corpus data at hand.

**FIGURE 11:**
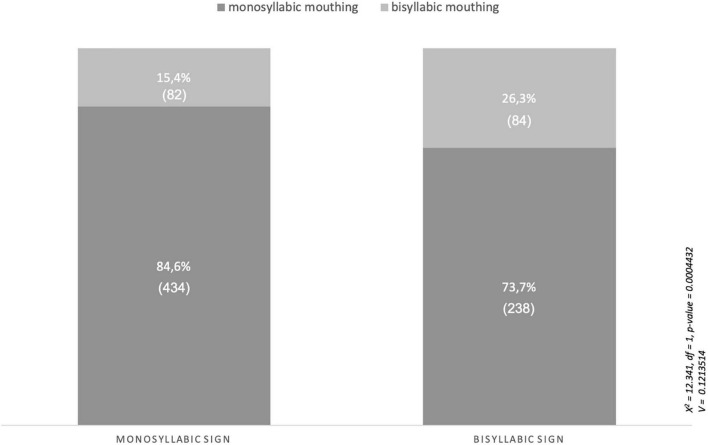
The number of monosyllabic and disyllabic RSL signs corresponding to monosyllabic and disyllabic mouthings.

For the above statistical analysis, each of the 30 signs that we evaluated (see Table 2) had been interpreted beforehand as mono- or disyllabic on the basis of the sign’s number of constitutive movements in the majority of its tokens. If, for example, a sign had more than 50% disyllabic tokens in the corpus, it was interpreted as a disyllabic sign in this analysis. It must be admitted, however, that some signs, such as WORK, CHEER or CHILD, appeared in the corpus as mono- or disyllabic in almost equal proportions. It is therefore of questionable validity to define them as being one or the other. With this drawback in mind, we supplement our statistical analysis by a detailed qualitative analysis of each token and find only a very weak relationship between a manual sign’s syllable structure and the number of co-occurring mouthed syllables. As Figure 11 shows, both mono- and disyllabic signs tended to pair with monosyllabic mouthings, namely in 84 and 73% of all cases, respectively. There was only a slightly heightened tendency for disyllabic mouthings to occur with disyllabic rather than monosyllabic signs. We measured the strength of the correlation between the variables by computing Cramér’s *V* (see Figure 11). The resulting value of 0.12 indicates a small effect size and gives an idea of the strength of the association within a range from 0 to 1. We interpret this association as being too weak to confirm the syllabic structure hypothesis (H1) and conclude that RSL signs prevailingly co-occur with monosyllabic mouthings irrespective of their syllabic structure.

Having found no significant correlation between the syllabic structure of manual signs and the reduction patterns of mouthings, we now turn to the second hypothesis. It posits that the stressed part of the spoken-language word is the part that is usually mouthed in sign languages (Bank, 2014, pp. 40–42). Up to now, the reduction of mouthing has been studied only for sign languages that are surrounded by Germanic spoken languages such as English, German or Dutch. The word-stress patterns of these West-Germanic spoken languages are very similar to each other (Domahs et al., 2014). Research shows that the potential default stress positions in German, English, and Dutch are the first (Levelt et al., 1999; Schiller et al., 2006) or the penultimate syllable (Eisenberg, 1991; Wiese, 1996).

Russian Sign Language is a sign language surrounded by a spoken language with variable stress. The position of the main stress in spoken Russian is largely unpredictable from a synchronic perspective. It can fall on a syllable in any position, depending on the word in question: e.g. on the first syllable, as in *prínter^13^* ‘printer,’ on the second syllable, as in *proféssor* ‘professor,’ on the third syllable, as in *inženér* ‘engineer’ etc. The stress is, moreover, movable and distinctive in the sense that different morphological forms of a lexeme may exhibit different syllable structures, as in (14).


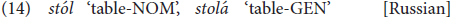


The position of stress in spoken Russian can also differentiate morphological forms, such as in *proféssora* ‘professor-GEN.SG’ vs. *professorá* ‘professor-PL.NOM’. RSL therefore lends itself well to investigating the potential relationship between the position of stress in a spoken word and the reduction pattern of a corresponding mouthing. The data in Table 2 suggest that reduced mouthings in RSL do not necessarily preserve the stressed syllable of the respective spoken Russian word. Our analysis of the 30 selected RSL signs (see Table 2) revealed that 17 signs occurred with mouthings containing only syllables that are not stressed in spoken Russian. Consider again the RSL sign MONKEY as an example (see Figure 10). The spoken Russian word for ‘monkey’ consists of four syllables and is always stressed on the third one (*o.bez’.já.na*). At the same time, more than 80% of this sign’s tokens in the RSL corpus were accompanied by a monosyllabic mouthing of the corresponding spoken word’s first syllable only, while the remaining 20% of sign tokens co-occurred with a mouthing of the respective word’s first and second syllable. Thus, the sign MONKEY was never accompanied by mouthing of the spoken word’s stressed syllable. Another pertinent example is the RSL sign STORE. The spoken Russian word ‘store’ consists of three syllables, and the stress falls on the third one (*ma.ga.zín*). The majority of this sign’s tokens (73%) again appeared with a monosyllabic mouthing of the spoken word’s first syllable *ma*. The remaining 27% of this sign’s tokens co-occurred with a disyllabic mouthing of the first two unstressed syllables. This is the predominant pattern suggested by our data, as shown in Table 2. Eighteen out of 30 RSL signs were accompanied by first-syllable mouthings in more than 50% of all cases while that same first syllable is not stressed in the corresponding spoken Russian words. Only six RSL signs (MOTHER, GIRL, WOOD, SMALL, CORRECT, CHEER) were accompanied by a first-syllable mouthing where that first syllable *is* stressed in the spoken Russian word. This appears to be a coincidence. Four RSL signs (CHILD, DOG, TRY, GOOD) co-occurred with mouthings that did not exclusively contain a stressed syllable. Consider, for instance, the RSL sign CHILD. It corresponds in spoken Russian to a trisyllabic word, which is stressed on the second syllable (*re.bë.nok*). The mouthings that accompanied this sign in the corpus reproduced only the first syllable in 45% of all cases, the first and second syllable in 40% of cases, only the second syllable in 5% of cases and the second and third syllable in 10% of cases.

The results of our data observation suggest that first syllables tend to be mouthed in RSL irrespective of the stress pattern of the corresponding word in the spoken language. In all 30 RSL signs under investigation, unstressed syllables were mouthed more often than stressed ones. To verify our initial observation, we tested the following hypothesis for the same 30 selected RSL signs:

*H1: If a syllable is stressed, it is mouthed in RSL.*

We added two new variables, “same_syl” and “diff_syl,” to the dataset (see Supplementary Table 2). The variable “same_syl” represented the percentage of cases where the same syllables visible in the RSL mouthing are stressed in spoken Russian. The variable “diff_syl” represents the percentage of cases where the syllables visible in the RSL mouthing are not stressed in spoken Russian. Because the data in these variables was not drawn from a normal distribution, we used the (Wilcoxon-) Mann–Whitney test. The *p*-value was below 0.05^14^, which means that the difference observable between the two variables was not due to chance. The above hypothesis (H1) thus could not be confirmed. A given syllable does not have to be visible in a mouthing just because its counterpart is stressed in the corresponding spoken Russian word. The syllables visible in RSL mouthings are most often unstressed.

Our analysis of reduced mouthings in RSL could not confirm the existing hypotheses for why only certain parts of a corresponding spoken word are mouthed in sign language. With regard to the structure of reduced mouthings, our study shows that they do not conform to the rhythm of the syllabic form of the co-occurring sign. Both monosyllabic and disyllabic signs tend to be accompanied by monosyllabic mouthings of the first syllable of the respective spoken Russian word. This finding suggests that signers are not drawing upon knowledge of the rhythmic structure of Russian words. The data show that the word-initial segments are the ones being retained in RSL reduced mouthings. One possibility is that signers rely on the written form and thus proceed from a representation of the beginning of a written word (This idea will be reinforced by our second finding, in “How Mouthings Differ From Spoken Russian Pronunciation,” below). We have furthermore shown that reduced mouthings do not mandatory reproduce the stressed part of the equivalent spoken language word, but rather tend to be constrained to the first syllable, irrespective of the stress pattern of the surrounding spoken language.

### How Mouthings Differ From Spoken Russian Pronunciation

Our investigation of mouthings in RSL has, so far, revealed that they do not always follow the phonological patterns of the spoken language and therefore cannot be regarded simply as borrowings of spoken language words or elements. In this section, we show that mouthings in RSL differ greatly from the observable pronunciation of spoken Russian words. This means that the lip movements of RSL mouthings differ from the lip movements of their spoken standard-Russian counterparts. As was already mentioned in “Study of Reduced Mouthings,” reduced mouthings are not systematically reduced to the stressed syllables of the spoken words. They are typically reduced to the first syllable or even the first segment of the respective spoken word, regardless of its stress pattern. This section shows that mouthings in RSL pattern more closely after written Russian. Our findings reveal that RSL mouthings do not exhibit vowel reduction patterns, which are obligatory present in spoken standard Russian.

Conforming to the prominent phonological feature of vowel reduction, unstressed vowels in Russian are pronounced differently to the same vowel phonemes in stressed position (Yanushevskaya and Bunčić, 2015). The unstressed non-high vowels /a/, /o/ and /e/ are reduced to [I] after soft onsets and to [a] elsewhere^15^, as illustrated by the examples in (15).


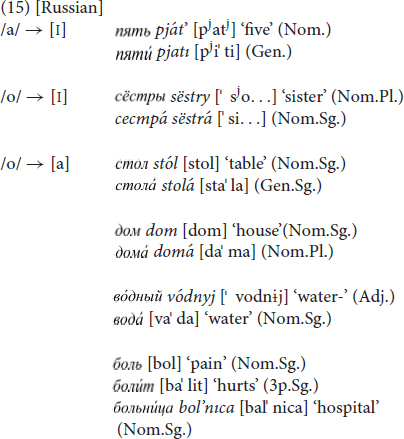


Because of the vowel reduction phenomenon, the orthographic representation and the pronunciation of vowels vary greatly from each other in Russian. Thus, although the words *vodnyj* and *voda* are both spelled with an *o*, the first syllable of the former is stressed and pronounced as [vo], while the first syllable of the latter is unstressed and thus pronounced as [va]. The difference between these vowels can easily be perceived upon the lips when pronounced: [a] is a central open unrounded vowel, [I] is a front closed unrounded vowel, and [o] is a half-closed rounded vowel.

**FIGURE 12:**
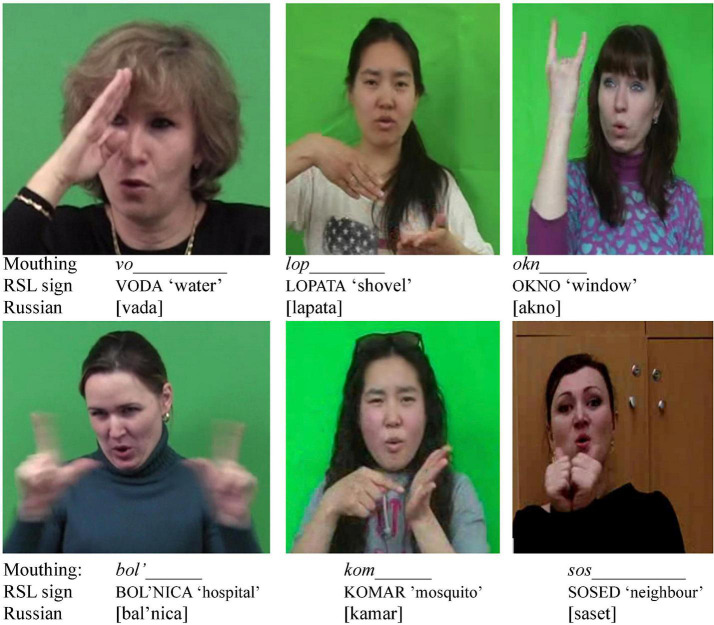
RSL signs and mouthings with rounded vowels. Reproduced with permission from Svetlana Burkova (Novosibirsk State Technical University), available at the online corpus of Russian Sign Language, http://rsl.nstu.ru/.

Research shows that lip rounding is the most easily visible labial feature. Both hearing and deaf people appear to be very good at visually perceiving such prominent features as the presence of lip rounding in vowels (Traunmüller and Öhrström, 2007). Assuming that signers are influenced only by visual information pertaining to lip movement in their production of mouthings, and given that they can easily detect the difference between the presence of lip rounding, as in [o], and the absence of lip rounding, as in [a], we can expect that the spoken Russian vowel reduction pattern would also be seen in mouthings. Thus, the sign VODA ‘water’ should be accompanied by mouthings with an unrounded vowel in the first syllable: namely [va] and not [vo], as is coded by orthographic *vo-*. Surprisingly, our corpus study shows a quite different pattern. RSL mouthings consistently fail to reproduce the vowel reduction patterns obligatory to the standard spoken Russian words that these mouthings are supposedly borrowings of. Thus, what we actually find is that the sign VODA ‘water’ is accompanied by a mouthing of [vo] in alignment with Russian orthography. It is a reduced mouthing with a rounded vowel, whereas the Russian pronunciation of the same word is [vaˈda] (featuring an unrounded vowel in the first syllable due to vowel reduction). Similarly, we observed in RSL mouthings numerous prominent visual cues indicating lip rounding in cases where there is no lip rounding of the corresponding spoken Russian vowels. Figure 12 presents images showing lip rounding in various RSL mouthings and a phonological transcription of the spoken Russian counterpart in contrast. The Russian pronunciations and the RSL mouthings evidently vary with regard to vowel reduction.

In all of the mouthings depicted in Figure 12, the lips were rounded. These examples are not exceptions. The RSL corpus exhibits numerous examples of mouthings that differ in this respect from standard Russian pronunciation. Supplementary Figure 1 shows that the six randomly chosen RSL signs most often co-occurred with mouthings containing a rounded vowel, which means they are not subject to vowel reduction patterns. The most variation was found across signers in mouthings of ‘water’ (full: [voˈda] ∼ reduced: [vaˈda]), although the mouthings in the RSL corpus nevertheless overwhelmingly lacked vowel reduction (i.e. [voˈda]). There are also numerous cases of full, i.e. unreduced, mouthings in the corpus that lack vowel reduction: e.g., *ogon’* [oˈgonʲ] ‘fire,’ *odežda* [o’d^j^eʒda] ‘clothes’ or *xotjat* [xoˈtʲat] ‘want.3PL’^16^. This finding suggests that the articulatory shape of mouthings in RSL is not likely under the influence of visual information pertaining to lip movements for equivalent words in the spoken Russian language. We therefore suggest that signers of RSL are more heavily influenced in their mouthing patterns by Russian orthography than by the visual information from lip movements in spoken Russian.

### Summary

This section presented our study of the most frequent type of mouthings in RSL, namely the reduced mouthing. We discussed the question of how and why mouthing reduction occurs in RSL and tested, based on our data, two hypotheses that have been postulated in the prior literature.

First, we found no statically significant correlation between the syllable structure of the manual sign (e.g., the number of its movements) and the form of the reduced mouthing (e.g., the number of its visible syllables), as has been hypothesized. Monosyllabic signs do not always occur with monosyllabic mouthings, and disyllabic signs are not even tendentially accompanied by disyllabic mouthings. Both mono- and disyllabic RSL signs tend to be accompanied by a monosyllabic mouthing. Reduced mouthings thus do not conform to the syllabic structure of the manual sign. Overall, our analysis suggests a tendency towards a monosyllabic mouthing. We did observe that disyllabic mouthings accompanied disyllabic signs more often than they did monosyllabic signs, but we found no strict relationship as would have confirmed the tested hypothesis.

Second, we scrutinized which of a spoken word’s syllables it is that are mouthed by RSL signers. We thereby challenged the hypothesis that mouthing always includes a stressed syllable, to which our data do not lend any support. In RSL, reduced mouthings do not necessarily reproduce the stressed part of the equivalent spoken Russian word, but rather tend to be constrained to the first syllable or even the first element of the word in question. In most cases, it was the first syllable of the respective lexical item that was reproduced. Accordingly, a significant number of signs in the RSL corpus were accompanied by mouthings containing syllables that are unstressed in spoken Russian.

Our further observations revealed that RSL mouthings differ in their articulatory appearance from the pronunciation of the equivalent elements in spoken standard Russian. RSL mouthings lack phonetic reduction of vowels, i.e. systematic changes in the acoustic quality of vowels as a result of the position of stress. Phonetic vowel reduction is obligatory present in spoken Russian and is therefore one of the sources of distinction between the spoken and written Russian language.

These findings have led us to re-think the origin of mouthing and additionally posit the written modality as one of its possible sources. We conclude based on the analyses presented in “Study of Reduced Mouthings” and “How Mouthings Differ From Spoken Russian Pronunciation” that the source for mouthings in RSL and, possibly in other sign languages as well, is a combination of the surrounding spoken and written language. Mouthings should therefore be considered not only a spoken- but also a written-language-based contact phenomenon.

## Discussion

Our corpus-based study offers new insights into the use of mouthings in RSL, in particular yielding interesting discoveries in terms of frequency rates, functions, distribution and spreading patterns, as well as the source of this cross-modal contact phenomenon. Specifically, our findings for RSL reveal quantitative differences between sign languages in the use of mouthings, which have previously been reported to comprise the most frequent category of all mouth actions in various sign languages (i.e., NGT or Auslan). A markedly different pattern was observed for RSL in this study. We could thus answer our first research question with respect to the frequency of mouthings by observing that they are just as frequent in the RSL corpus as are mouth gestures, at a rate of 44%. This confirms the intuitions of the RSL signers who reported to us that they use fewer mouthings in their RSL signing than they do in other sign languages. Based on this finding and given the cross-linguistic comparability of our data (a large corpus, various text-types, similar annotations, analogous data analysis), we were able to conclude that sign languages differ in terms of the prevalence of mouthings. Our findings additionally suggest that sign languages also systematically differ with respect to the form of their mouthings. A recent STS corpus study showed that less than a quarter of all mouthings were reduced (20%). We have shown that mouthings in RSL appear mainly in their reduced form (75%). Thus, the use of mouthing types (full vs. reduced) differs cross-linguistically. The differences between the frequency of reduced forms of mouthing in RSL and STS might relate to the relative morphological complexity of spoken Russian in contrast to the relative poverty of inflectional morphology in spoken Swedish^17^. This idea should be investigated by future studies.

Our analysis of the distribution of mouthings in relation to grammatical class and sign type in many ways confirmed earlier findings reported for other sign languages. It was shown that mouthings in RSL most frequently accompany function words (auxiliaries, prepositions, conjunctions and *wh*-question words), numbers and nouns. Spatial verbs, discourse markers, interjections, negators and locatives most strongly disfavor mouthings and more readily co-occur with mouth gestures or with no mouth action at all.

Our findings lend support to those of Johnston et al. (2016) in that mouthings differ greatly over various sign types. Our data suggest that mouthing may be an obligatory formational component of fingerspelling, as fingerspelled elements in RSL were shown to co-occur with mouthings in 98% of all cases. This result is also in line with previous studies. We furthermore expanded our scope to include SASSes, which have not been analyzed in conjunction with mouthings in earlier research. By contrasting fingerspelled elements with SASSes, we showed that the use of mouthings in RSL can really only be properly considered in relation to various sign types. While fingerspellings almost always co-occur with mouthings, SASSes only rarely do so. This finding additionally underlines the strength of large corpus data for investigating mouthings.

Beyond the frequency and distribution of mouthings, we explored their spreading patterns in RSL for the first time. The results presented here show similarities with other sign languages in the prevalence of progressive spreading. However, regressive spreading was shown to be possible in RSL when the mouthing spreads over a pointing sign. In addition to such standardly observable spreading from a (prototypically content-expressing) sign to one or more adjacent (prototypically functional) signs, we encountered spreading of free mouthings, as well as spreading that extend away from functional signs. These non-prototypical patterns are possible when a spreading connects two phrases, thus contributing to the coherence of the discourse.

Our second research question concerned the functions performed by mouthings. Based on our analysis, we were able to identify and describe a new function, namely disambiguation between different sign types. A mouthing performs this function when it co-occurs with a manual item that can otherwise be interpreted as either a fully-lexicalized or a partially-lexicalized sign, depending on its context. The co-occurrence of a mouthing together with the manual item functions as one of the indicators that it should be interpreted as a fully-lexicalized sign.

Pursuant to our third research question, we explored how and why mouthing reduction occurs in RSL. Our comprehensive analysis did not confirm the syllabic-structure hypothesis. Against anticipations, mouthings do not conform to the rhythm of the syllabic structure of accompanying manual RSL signs. We found that RSL signs prefer monosyllabic mouthings irrespective of their own syllabic structure.

The hypothesis that the stressed part of a spoken word is always reproduced in mouthings also could not be confirmed. Our data revealed that reduced mouthings in RSL do not systematically reproduce the stressed part of the equivalent spoken language word. We found instead that reduced mouthings mainly consist of the first syllable, irrespective of the stress pattern observable in spoken Russian. In addition, our study revealed that Russian pronunciation and RSL mouthings vary with respect to vowel reduction patterns and that, in this respect, mouthings pattern after Russian orthography more closely than the pronunciation of spoken Russian.

Given our results, and contrary to earlier assumptions that mouthings originate as borrowings of words from a surrounding spoken language through the observation of speakers’ lip movements, we suggest that RSL signers are influenced in their mouthing production also by written language. We suspect that other sign languages may not be very different in this regard, but we must await further research on languages in which spelling and pronunciation do not correspond in a predictable way. Moreover, studies on the acquisition of mouthings^18^ by preschool children prior to their exposure to the written language are also necessary in order to fully support our thesis. It is possible that children learn to mouth twice in their life, as has been suggested by Padden (2005) with respect to fingerspelling. First, a child may learn to use mouthing patterns as whole units, mimicking the lip movements of other signers or speakers. Later, when reading and writing become more prominent in everyday life, the child begins to understand mouthings as being made up of articulations that correspond to the letters of the alphabet. Accordingly, the child then learns mouthings a second time – this time in close connection to words in their written form.

Surely, some examples contradicting this claim can be found. Not all RSL mouthings lack the vowel reduction patterns of spoken Russian. Some high-frequency signs (e.g. WATER) do, at times, occur with vowel reduction features evident in the lip movements (see Supplementary Figure 1). This might vary from signer to signer based on the individual’s proficiency in speechreading, their knowledge of the structure of linguistic sounds and how they are articulated in speech, their amount of exposure to, and the quality of, oral education and possibly other factors, which are yet to be investigated. Written language is an inevitable part of life for deaf people (at least in literate communities with access to formal education). Moreover, all signers display some degree of bilingualism, so we estimate that the impact of the orthographic representation of words is higher than that which occurs from observation of the lip movements performed in the speech of hearing people. In DGS, a similar mismatch between German pronunciation and DGS mouthings can be observed. Consider the examples of *Iphone* or *Dublin*. In the DGS Corpus, we can find the mouthings [ifon] and [dublin], which differ from the standard German pronunciations [ˈaɪˌfəʊn] and [ˈdʌblɪn]. Thus, with respect to the possibility that written language may serve as a source of the linguistic content drawn upon in mouthings, we propose that signers are guided in the articulation of their mouthings by both the orthographic form of a corresponding word and its pronunciation. Mouthings thereby reflect the constant situation of language contact between sign languages and their surrounding written and spoken languages, whereby the impact that a written language may have upon a sign language can evidently be stronger than previously assumed.

To conclude, the results of this quantitative corpus-based study contribute to our general understanding of mouthings and reveal that the multimodal practices of deaf signers are predicated upon a more complex interplay of signed, spoken, and written languages than has previously been thought.

## Data Availability Statement

The datasets presented in this study can be found in online repositories. The names of the repository/repositories and accession number(s) can be found in the article/Supplementary Material.

## Ethics Statement

The studies involving human participants were reviewed and approved by German Science Foundation. The patients/participants provided their written informed consent to participate in this study. Written informed consent was obtained from the individual(s) for the publication of any potentially identifiable images or data included in this article.

## Author Contributions

AB: methodology, corpus investigation, writing of the sections “Frequency of Mouthings,” “Distribution Over Grammatical Class and Sign Type,” and “New Insights Into the Origin of Mouthings” and acquisition of funding. MK: methodology, elicitation analysis, and writing of the sections “Spreading Patterns” and “New insights into the functions of mouthings”. AB and MK: conceptualization and writing the remaining sections. Both authors: conceptualization, analysis, writing and editing of this manuscript, read and agreed to the published version of the manuscript.

## Conflict of Interest

The authors declare that the research was conducted in the absence of any commercial or financial relationships that could be construed as a potential conflict of interest.

## Publisher’s Note

All claims expressed in this article are solely those of the authors and do not necessarily represent those of their affiliated organizations, or those of the publisher, the editors and the reviewers. Any product that may be evaluated in this article, or claim that may be made by its manufacturer, is not guaranteed or endorsed by the publisher.

## Footnotes

1 We follow the convention of using “Deaf” to refer to sign language users who are also part of a cultural minority. Uncapitalized “deaf” is used to refer solely to audiological status or in cases where linguistic and cultural status is not being highlighted.

2 The segments in brackets are not mouthed.

3 Meaning it is visibly articulated by the signer.

4 Russ. *likvidacija bezgrammotnosti* ‘elimination of illiteracy’.

5 Another attested spelling of this name in English is *Zaitseva*.

6
http://rsl.nstu.ru/

7 In the numerous cases where the categories overlap (e.g., reduced and inflected mouthing overlap often, as well as full and variant mouthing etc.), both were annotated.

8 One example of such debatable cases is a mouth movement *shhhhh* [ʂː] produced by some RSL signers in the corpus together with the manual sign ŠKOLA ‘school,’ which starts with a postalveolar fricative in spoken Russian. The Deaf native RSL signer was sure to annotate it as a mouthing, whereas the hearing assistant wanted to mark it as a mouth gesture. Because this mouth action accompanied a morphologically simple sign, we decided to annotate it as a mouthing in the RSL corpus.

9 Cases of polysemous signs differentiated by mouthing were also included in the category of variant mouthing.

10 We are aware of the unique status that fingerspelled items have in the sign language lexicon and of the potential implications that this status has on their interaction with mouthing. They violate the phonological structure of the native lexicon (which SASSes do not). Nevertheless, following Johnston et al. (2016), we consider fingerspelling to be a separate sign type.

11 It is important to keep in mind, however, that more-lexicalized SASSes act as nouns in a sentence, whereas less-lexicalized SASSes prototypically act as predicate adjectives. Therefore, one could argue that mouthing here has an already established role as a mechanism that helps to distinguish between nominal and predicate signs. In order to instead confirm our competing hypothesis that mouthing in these examples differentiates between fully- and partially-lexicalized signs, additional data from sign language classifier constructions are needed. In the case of classifier constructions, the change of the status from less- to more-lexicalized does not lead to a change of the sign’s part of speech. Therefore, a more frequent co-occurrence of mouthings with fully-lexicalized classifier constructions would serve as evidence in favor of our hypothesis.

12 These 30 RSL signs were the most frequent ones among RSL signs with at least a three-syllable citation form in spoken Russian.

13 We follow the Russistic convention of marking stress with an acute. Syllables bearing the letter ⟨ë⟩ also carry the stress.

14 *p* = 5.021e-08.

15 It is worth stating for the benefit of critical readers familiar with Russian phonology that this is of course a simplified explanation of the phenomenon.

16 This observation provides even more support for our thesis. Otherwise, one could entertain another possibility. Signers of RSL could have internalized the phonological rules of spoken Russian vowel reduction (given their heightened lip-reading ability) such that they would then apply them within their own reduced mouthings. So, for example, any monosyllabic mouthing (e.g. *sos(ed)* ‘neighbor,’ reduced to *sos*) with an underlying /o/ would necessarily be produced with a rounded [o] because monosyllabic prosodic units are always stressed in Russian (e.g. *stol* [stol] ‘table’). To produce a form with [a] in such a case would be counter to the laws governing vowel reduction. This requires, however, that the visible segments would have been (re)processed by signers as a complete prosodic unit, in addition to their having internalized the rules of Russian vowel reduction. The fact that we find instances in which unreduced mouthings fail to exhibit vowel reduction patterns (e.g. a mouthing that resembles [oˈgonʲ] rather than [aˈgonʲ]) overrides both assumptions.

17 We thank one of our reviewers for this suggestion.

18 Studies on the acquisition of mouthings are virtually nonexistent (but see a recent study by Woll et al., 2019).
