# Supplementary material for: New Insights Into Mouthings: Evidence From a Corpus-Based Study of Russian Sign Language
Source: Front Psychol. 2022 Feb 22;12:779958. doi: 10.3389/fpsyg.2021.779958 (PMC8904218; doi:10.3389/fpsyg.2021.779958)
Supplement: Supplementary file 1 [file Table_1.DOCX]

**Supplementary Table 1: Grammatical class & mouthing**

| **Part of Speech** | **Log Odd** | **Tokens** | **% with mouthing** |
| --- | --- | --- | --- |
| Auxiliary | 3.04452 | 22 | 95.5 |
| Preposition | 2.83321 | 18 | 94.5 |
| Conjunction | 2.56949 | 28 | 92.9 |
| Numeral | 1.52605 | 56 | 82.1 |
| Noun | 1.05860 | 462 | 74.2 |
| WH-word* | 0.79850 | 29 | 69 |
| Adjective | 0.42199 | 101 | 60.4 |
| Adverb* | 0.34484 | 205 | 58.5 |
| PRO | -0.18775 | 203 | 45.3 |
| Determiner | -0.35668 | 34 | 41 |
| Verb | -0.36735 | 501 | 40.9 |
| Agreeing verb | -0.59784 | 31 | 35.4 |
| Spatial verb | -0.94739 | 68 | 27.9 |
| Discourse marker | -1.14939 | 133 | 24.1 |
| Interjection | -1.88707 | 38 | 13.2 |
| Negator | -1.94591 | 8 | 12.5 |
| Locative | -2.18123 | 69 | 10.1 |

*All factor groups significant at p < .05, with the exception of WH-word and adverb.
