# Supplementary material for: New Insights Into Mouthings: Evidence From a Corpus-Based Study of Russian Sign Language
Source: Front Psychol. 2022 Feb 22;12:779958. doi: 10.3389/fpsyg.2021.779958 (PMC8904218; doi:10.3389/fpsyg.2021.779958)
Supplement: Supplementary file 2 [file Table_2.docx]

|  | English translation | Russian word stress | RSL sign annotation | spoken Russian  accent | mono-syllabic sign | bi-syllabic sign | full mouthing | 1^st^ part of the word | 1^st^-2^nd^ syl | 2^nd^ syl | 2^nd^-3^rd^ syl | 3^rd^ syl | 1^st^-3^rd^ syl | same_syl | diff_syl | mouthed_1 | mouthed_2 |
| --- | --- | --- | --- | --- | --- | --- | --- | --- | --- | --- | --- | --- | --- | --- | --- | --- | --- |
| 1 | GRANDMOTHER | 1 | БАБУШКА 'grandmother' (n=41) | 1 | 0.63 | 0.28 | 0.1 | 0.59 | 0.13 | 0 | 0 | 0 | 0 | 0.59 | 0.41 | 0.59 | 0.23 |
| 2 | SPEAK | 3 | ГОВОРИТЬ 'to speak' (n=21) | 3 | 0.57 | 0.3 | 0.05 | 0.48 | 0.28 | 0.14 | 0 | 0.05 | 0 | 0.05 | 0.95 | 0.67 | 0.33 |
| 3 | GIRL | 1 | ДЕВУШКА 'girl' (n=64) | 1 | 0.61 | 0.36 | 0 | 0.74 | 0.23 | 0.03 | 0 | 0 | 0 | 0.74 | 0.26 | 0.77 | 0.23 |
| 4 | WOOD | 1 | ДЕРЕВО 'wood' (n=19) | 1 | 0.71 | 0.29 | 0 | 0.6 | 0.4 | 0 | 0 | 0 | 0 | 0.6 | 0.4 | 0.6 | 0.4 |
| 5 | INTERESTING | 3 | ИНТЕРЕСНЫЙ 'interesting' (n=38) | 3 | 0.76 | 0.24 | 0 | 0.39 | 0.33 | 0 | 0 | 0 | 0.28 | 0 | 1 | 0.39 | 0.61 |
| 6 | COMPUTER | 2 | КОМЬЮТЕР 'computer' (n=22) | 2 | 0.43 | 0.57 | 0 | 0.72 | 0.28 | 0 | 0 | 0 | 0 | 0 | 1 | 0.72 | 0.28 |
| 7 | BEAUTIFUL | 2 | КРАСИВЫЙ 'beautiful' (n=19) | 2 | 0.95 | 0.05 | 0 | 0.5 | 0.28 | 0.05 | 0.11 | 0.06 | 0 | 0.05 | 0.95 | 0.61 | 0.39 |
| 8 | STORE | 3 | МАГАЗИН 'store' (n=22) | 3 | 0.77 | 0.18 | 0 | 0.73 | 0.27 | 0 | 0 | 0 | 0 | 0 | 1 | 0.73 | 0.27 |
| 9 | SMALL | 1 | МАЛЕНЬКИЙ 'small' (n=63) | 1 | 0.95 | 0.05 | 0 | 0.85 | 0.05 | 0.1 | 0 | 0 | 0 | 0.85 | 0.15 | 0.95 | 0.05 |
| 10 | MAN | 2 | МУЖЧИНА 'man' (n=46) | 2 | 0.57 | 0.37 | 0 | 0.92 | 0.04 | 0 | 0.04 | 0 | 0 | 0 | 1 | 0.92 | 0.08 |
| 11 | FOR-EXAMPLE | 3 | НАПРИМЕР 'for example' (n=93) | 3 | 0.33 | 0.65 | 0 | 0.78 | 0.05 | 0.11 | 0.04 | 0.02 | 0 | 0.02 | 0.98 | 0.91 | 0.09 |
| 12 | NOVOSIBIRSK | 4 | НОВОСИБИРСК 'Novosibirsk' (n=22) | 4 | 0.23 | 0.77 | 0 | 0.63 | 0.17 | 0.12 | 0 | 0 | 0 | 0 | 1 | 0.75 | 0.17 |
| 13 | NORMAL | 2 | НОРМАЛЬНЫЙ 'normal' (n=22) | 2 | 0.59 | 0.23 | 0 | 0.59 | 0.41 | 0 | 0 | 0 | 0 | 0 | 1 | 0.59 | 0.41 |
| 14 | MONKEY | 3 | ОБЕЗЬЯНА 'monkey' (n=21) | 3 | 0.33 | 0.67 | 0 | 0.8 | 0.2 | 0 | 0 | 0 | 0 | 0 | 1 | 0.8 | 0.2 |
| 15 | RETUREN | 2 | ОБРАТНО 'return' (n=39) | 2 | 0.82 | 0.18 | 0 | 0.64 | 0.31 | 0.05 | 0 | 0 | 0 | 0.05 | 0.95 | 0.69 | 0.31 |
| 16 | COMMUNICATE | 2 | ОБЩАТЬСЯ 'communicate' (n=25) | 2 | 0.44 | 0.39 | 0.14 | 0.76 | 0.1 | 0 | 0 | 0 | 0 | 0 | 1 | 0.76 | 0.24 |
| 17 | CLASSMATE | 3 | ОДНОКЛАССНИК 'classmate' (n=12) | 3 | 0.75 | 0.25 | 0 | 1 | 0 | 0 | 0 | 0 | 0 | 0 | 1 | 1 | 0 |
| 18 | TENT | 2 | ПАЛАТКА 'tent' (n=24) | 2 | 1 | 0 | 0 | 0.67 | 0.33 | 0 | 0 | 0 | 0 | 0 | 1 | 0.67 | 0.33 |
| 19 | HELP | 3 | ПОМОГАТЬ 'help' (n=33) | 3 | 0.21 | 0.73 | 0 | 0.64 | 0.36 | 0 | 0 | 0 | 0 | 0 | 1 | 0.64 | 0.36 |
| 20 | CORRECT | 1 | ПРАВИЛЬНЫЙ 'correct' (n=23) | 1 | 1 | 0 | 0 | 1 | 0 | 0 | 0 | 0 | 0 | 1 | 0 | 1 | 0 |
| 21 | WORK | 2 | РАБОТАТЬ 'work' (n=41) | 2 | 0.42 | 0.51 | 0 | 0.77 | 0.15 | 0 | 0 | 0 | 0 | 0 | 1 | 0.77 | 0.15 |
| 22 | CHEER | 1 | РАДОВАТЬСЯ 'cheer' (n=24) | 1 | 0.46 | 0.42 | 0 | 0.4 | 0.27 | 0.07 | 0 | 0.13 | 0.13 | 0.4 | 0.6 | 0.6 | 0.4 |
| 23 | CHILD | 2 | РЕБЕНОК 'child' (n=30) | 2 | 0.53 | 0.42 | 0 | 0.45 | 0.4 | 0.05 | 0.1 | 0 | 0 | 0.05 | 0.95 | 0.5 | 0.5 |
| 24 | DOG | 2 | СОБАКА 'dog' (n=48) | 2 | 0.38 | 0.58 | 0.15 | 0.38 | 0.32 | 0.15 | 0 | 0 | 0 | 0.15 | 0.85 | 0.53 | 0.47 |
| 25 | CALM | 2 | СПОКОЙНЫЙ 'calm' (n=18) | 2 | 1 | 0 | 0 | 0.88 | 0.12 | 0 | 0 | 0 | 0 | 0 | 1 | 0.88 | 0.12 |
| 26 | TRY | 2 | СТАРАТЬСЯ 'to try' (n=17) | 2 | 0.76 | 0.24 | 0.06 | 0.41 | 0.29 | 0.18 | 0 | 0.06 | 0 | 0.18 | 0.82 | 0.65 | 0.35 |
| 27 | COLD | 2 | ХОЛОДНЫЙ 'cold' (n=17) | 2 | 0.23 | 0.53 | 0 | 1 | 0 | 0 | 0 | 0 | 0 | 0 | 1 | 1 | 0 |
| 28 | GOOD | 2 | ХОРОШИЙ 'good' (n=42) | 2 | 0.86 | 0.12 | 0.09 | 0.19 | 0.24 | 0.26 | 0.11 | 0.02 | 0 | 0.26 | 0.74 | 0.47 | 0.44 |
| 29 | PERSON | 3 | ЧЕЛОВЕК 'person' (n=20) | 3 | 1 | 0 | 0.41 | 0.59 | 0 | 0 | 0 | 0 | 0 | 0 | 1 | 0.59 | 0.41 |
| 30 | FEEL | 1 | ЧУВСТВОВАТЬ 'to feel' (n=35) | 1 | 0.57 | 0.43 | 0 | 0.94 | 0.06 | 0 | 0 | 0 | 0 | 0.94 | 0.06 | 0.94 | 0.06 |

**Supplementary Table 2. Data for the RSL reduction study**
