# Supplementary figures and images for: New Insights Into Mouthings: Evidence From a Corpus-Based Study of Russian Sign Language
Source: Front Psychol. 2022 Feb 22;12:779958. doi: 10.3389/fpsyg.2021.779958 (PMC8904218; doi:10.3389/fpsyg.2021.779958)

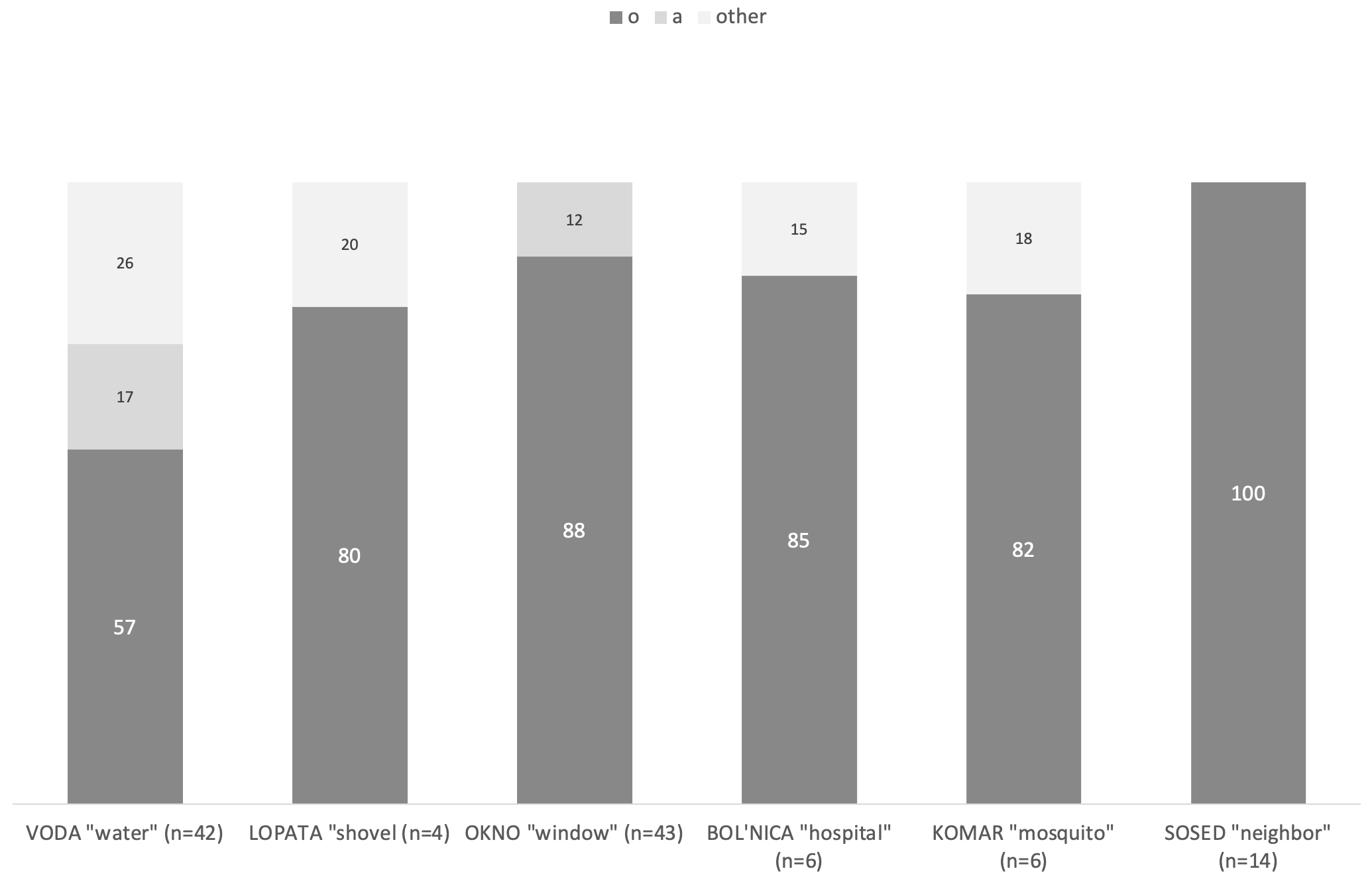

Supplement: Supplementary file 3 [file Image_1.png]
